# The ESCRT-III Protein CHMP1A Mediates Secretion of Sonic Hedgehog on a Distinctive Subtype of Extracellular Vesicles

**DOI:** 10.1016/j.celrep.2018.06.100

**Published:** 2018-07-24

**Authors:** Michael E. Coulter, Cristina M. Dorobantu, Gerrald A. Lodewijk, François Delalande, Sarah Cianferani, Vijay S. Ganesh, Richard S. Smith, Elaine T. Lim, C. Shan Xu, Song Pang, Eric T. Wong, Hart G.W. Lidov, Monica L. Calicchio, Edward Yang, Dilenny M. Gonzalez, Thorsten M. Schlaeger, Ganeshwaran H. Mochida, Harald Hess, Wei-Chung Allen Lee, Maria K. Lehtinen, Tomas Kirchhausen, David Haussler, Frank M.J. Jacobs, Raphael Gaudin, Christopher A. Walsh

**Affiliations:** 1 Division of Genetics and Genomics and Howard Hughes Medical Institute, Boston Children’s Hospital, Departments of Pediatrics and Neurology, Harvard Medical School, Boston, MA 02115, USA; 2 Program in Neuroscience and Harvard/MIT MD-PHD Program, Harvard Medical School, Boston, MA 02115, USA; 3 Inserm U1110, Université de Strasbourg, Institut de Recherche sur les Maladies Virales et Hépatiques, 67000 Strasbourg, France; 4 University of Amsterdam, Swammerdam Institute for Life Sciences, 1098 XH Amsterdam, the Netherlands; 5 Laboratoire de Spectrométrie de Masse Bio-Organique, IPHC, UMR 7178, CNRS-Université de Strasbourg, ECPM, 67087 Strasbourg, France; 6 Department of Neurology, Massachusetts General Hospital, Boston, MA 02114, USA; 7 Department of Neurology, Brigham and Women’s Hospital, Boston, MA 02115, USA; 8 Janelia Research Campus, Howard Hughes Medical Institute, Ashburn, VA 20147, USA; 9 Brain Tumor Center and Neuro-Oncology Unit, Beth Israel Deaconess Medical Center, Boston, MA 02215, USA; 10 Department of Pathology, Boston Children’s Hospital, Boston, MA 02115, USA; 11 Department of Radiology, Boston Children’s Hospital, Boston, MA 02115, USA; 12 Division of Hematology and Oncology, Boston Children’s Hospital, Boston, MA 02115, USA; 13 F.M. Kirby Neurobiology Center, Boston Children’s Hospital and Department of Neurology, Harvard Medical School, Boston, MA 02115, USA; 14 Program in Cellular and Molecular Medicine, Boston Children’s Hospital and Department of Cell Biology, Harvard Medical School, Boston, MA 02115, USA; 15 Department of Pediatrics, Harvard Medical School, Boston, MA 02115, USA; 16 Center for Biomolecular Science and Engineering, University of California and Howard Hughes Medical Institute, Santa Cruz, CA 95064, USA; 17 These authors contributed equally; 18 Lead Contact

## Abstract

Endosomal sorting complex required for transport (ESCRT) complex proteins regulate biogenesis and release of extracellular vesicles (EVs), which enable cell-to-cell communication in the nervous system essential for development and adult function. We recently showed human loss-of-function (LOF) mutations in ESCRT-III member *CHMP1A* cause autosomal recessive microcephaly with pontocerebellar hypoplasia, but its mechanism was unclear. Here, we show *Chmp1a* is required for progenitor proliferation in mouse cortex and cerebellum and progenitor maintenance in human cerebral organoids. In *Chmp1a* null mice, this defect is associated with impaired sonic hedgehog (Shh) secretion and intraluminal vesicle (ILV) formation in multivesicular bodies (MVBs). Furthermore, we show *CHMP1A* is important for release of an EV subtype that contains AXL, RAB18, and TMED10 (ART) and SHH. Our findings show *CHMP1A* loss impairs secretion of SHH on ART-EVs, providing molecular mechanistic insights into the role of ESCRT proteins and EVs in the brain.

## In Brief

Extracellular vesicles (EVs) are essential for cell-to-cell communication in developing brain. Coulter et al. show that the human microcephaly gene *CHMP1A* is required for neuroprogenitor proliferation through regulation of vesicular secretion of the growth factor sonic hedgehog (SHH). *CHMP1A* specifically impairs SHH secretion on a distinctive EV subtype, ART-EV.

## Graphical Abstract

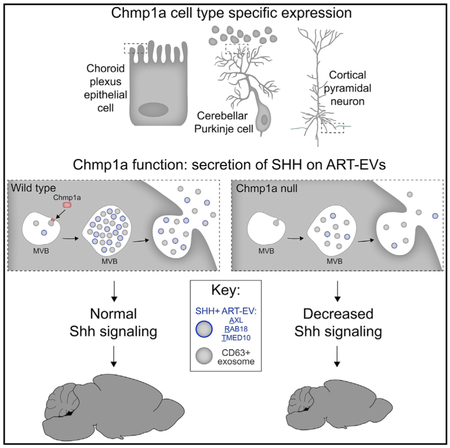

## INTRODUCTION

Extracellular vesicles (EVs) are increasingly recognized as essential mediators of specialized cellular secretion, but the mechanisms of EV function are not well understood, partly because of the diversity of EV subtypes (Bobrie et al., 2012; Kowal et al., 2016) and the lack of tools to specifically disrupt individual EV subtypes. EVs are essential for cell-to-cell communication, allowing hydrophobic signaling molecules (Korkut et al., 2009), RNA (Tietje et al., 2014), and other specialized cargo (Budnik et al., 2016) to travel through an aqueous extracellular environment. At the *Drosophila* neuromuscular junction, EV-mediated wingless secretion is required for synapse growth, EV-mediated Synaptotagmin 4 secretion is required for retrograde signaling, and EV-mediated transfer of Arc1 is required for synapse maturation (Koles et al., 2012; Korkut et al., 2013; Ashley et al., 2018). Cultured mammalian neurons (Lachenal et al., 2011), oligodendrocytes (Frühbeis et al., 2013), and microglia (Antonucci et al., 2012) secrete EVs, and recent work showed EVs play an active role in synaptic plasticity by mediating neuron-to-neuron transfer of *Arc* mRNA, a master regulator of activity-dependent glutamate receptor trafficking (Pastuzyn et al., 2018). There is also evidence that EVs may mediate pathological transfer of prion-like proteins and Tau (Asai et al., 2015). However, these functions in mammalian neurons remain somewhat speculative because of a lack of *in vivo* vertebrate models that selectively disrupt EV function.

Sonic hedgehog (Shh) is a hydrophobic secreted factor essential for embryonic development, serving as a morphogen (Cohen et al., 2015; Roelink et al., 1995), a mitogen (Nielsen and Dymecki, 2010; Dahmane and Ruiz i Altaba, 1999), an axon guidance molecule (Wilson and Stoeckli, 2013; Charron et al., 2003), and a regulator of synapse formation (Harwell et al., 2012). In developing cerebellum, Shh stimulates proliferation of granule cell precursors (GCPs), progenitor cells that generate granule neurons, the most abundant neuron in the brain (Zhou et al., 2007); because of this role, loss of *Shh* causes profound cerebellar hypoplasia (Corrales et al., 2006). Whereas the source of secreted Shh that regulates GCP proliferation is Purkinje cells (PCs) (Wechsler-Reya and Scott, 1999), the mechanism of Shh secretion is unclear, because studies have reported multiple different secretion mechanisms, including oligomeric complexes (Zeng et al., 2001), lipoprotein particles (Panáková et al., 2005), and EVs (Matusek et al., 2014; (Vyas et al., 2014).

Endosomal sorting complex required for transport (ESCRT) machinery regulates EV formation and release, as well as other membrane remodeling processes in the cell. ESCRT members are grouped into four subunits (0–III) that drive different steps in membrane remodeling, including deformation, budding, and scission (McCullough et al., 2013). The ESCRT-III complex comprises eleven subunits designated CHMPs (charged multivesicular body proteins) that are particularly important for membrane scission. Several conflicting potential mechanisms have been proposed to explain why loss-of-function (LOF) mutations in an ESCRT-III member, *CHMP1A*, cause microcephaly with pontocerebellar hypoplasia and short stature in humans (Mochida et al.,2012; Howard et al., 2001). Here, we show that, analogous to *Drosophila* (Matusek et al., 2014), *CHMP1A* is an essential mediator of vertebrate SHH secretion during brain development. *Chmp1a* null mice show widespread defects in forebrain and hindbrain development with evidence of disrupted *Shh* signaling, which can be rescued by activation of downstream signaling. Furthermore, the Shh protein level in the cerebrospinal fluid (CSF) of *Chmp1a* null embryos is markedly reduced compared to littermate controls. *Chmp1a* is specifically required for vesicular SHH secretion. *Chmp1a* loss impairs EV biogenesis by reducing intraluminal vesicles (ILVs) within multivesicular bodies (MVBs) and disrupts secretion of a distinctive SHH-positive EV subtype we call ART-EV (AXL, RAB18, and TMED10 extracellular vesicle). The *Chmp1a* null mouse highlights the diversity of EV subtypes but also provides a crucial vertebrate model to dissect the various functions of EVs in the nervous system.

## RESULTS

### *Chmp1a* Is Required for Embryonic Development

We created a *Chmp1a* gene trap (GT) mouse line (Figure S1) that removes Chmp1a protein. A GT cassette (Stryke et al., 2003) inserted in intron 1 of *Chmp1a* contains a strong splice acceptor from *En2* fused to the coding sequence for β-galactosidase (Figure S1A). Embryonic stem cells (ESCs) containing this GT allele were injected into mouse blastocysts to generate *Chmp1a* GT chimeras, which were outcrossed to create germline *Chmp1a* GT allele carriers. DNA sequencing confirms that in homozygous GT embryos, *Chmp1a* intron 1 is fused to *En* intron 1 (Figure S1C). Heterozygous GT mice show reduced Chmp1a protein expression compared to wild-type (WT), and homozygous GT mice express no detectable Chmp1a protein (Figure S1B), confirming a null mutation.

**Figure 1.**
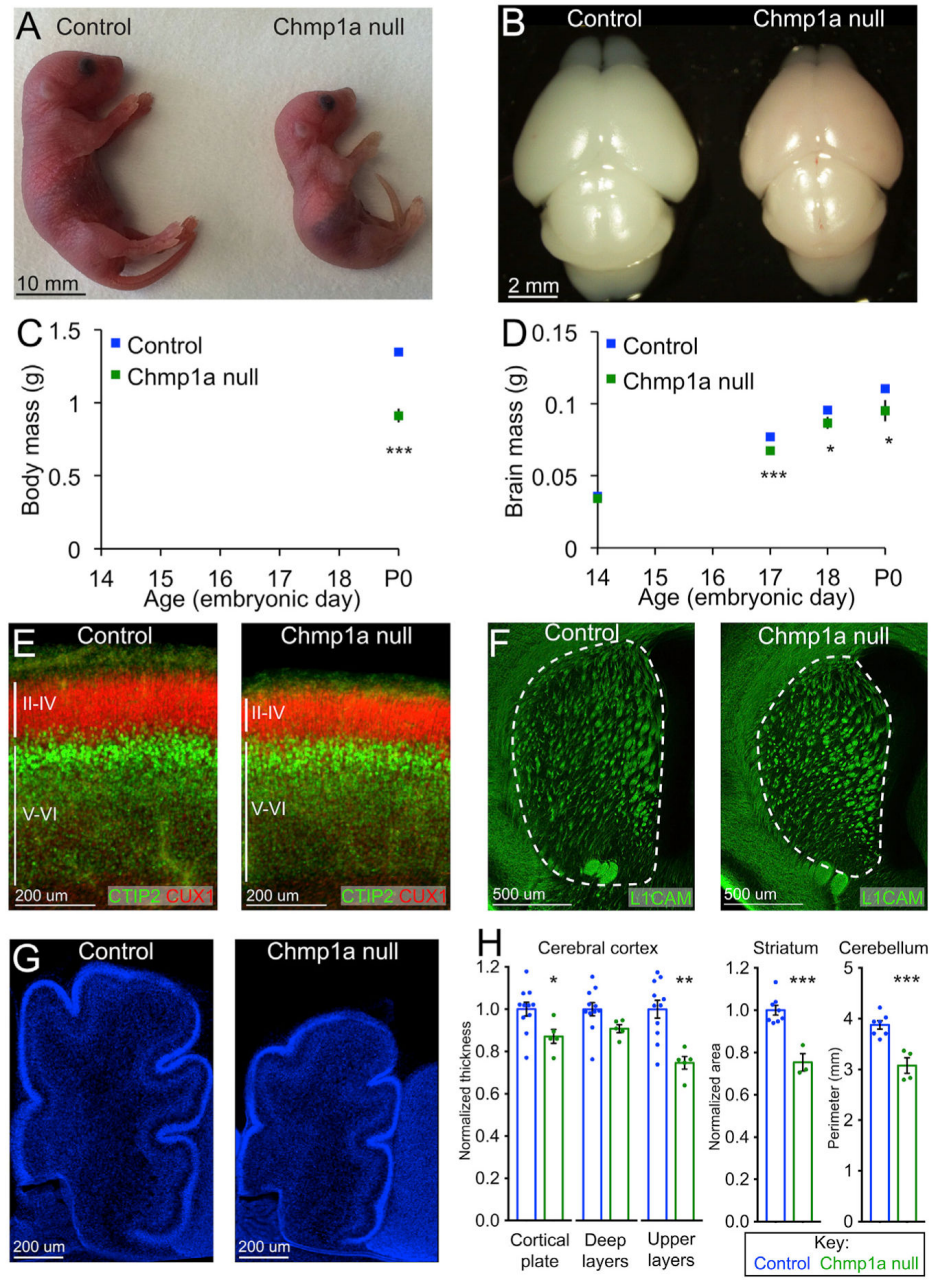
*Chmp1a* Is Essential for Brain Development in Mice (A) P0 *Chmp1a* null pups are smaller than litter-mate controls. (B) P0 *Chmp1a* null pups have smaller brains than controls. Note smaller olfactory bulbs and shorter anterior-posterior (A-P) length of the cortex. (C) Body mass is reduced in *Chmp1a* null embryos. P0: control, n = 19; *Chmp1a* null, n = 3. (D) Brain mass is reduced in *Chmp1a* null embryos. E14.5: control, n = 10; *Chmp1a* null, n = 5. E17.5: control, n = 23; *Chmp1a* null, n = 4. E18.5: control, n = 20; *Chmp1a* null, n = 3. P0: control, n = 9; *Chmp1a* null, n = 3. (E) Cortical plate is 13% thinner in *Chmp1a* null embryos at E18.5. Control, n = 11; *Chmp1a* null, n = 5. (F) Striatum area is reduced by 25% at E18.5 in *Chmp1a* null embryos compared to controls. Control, n = 8; *Chmp1a* null, n = 3. (G) P0 midline section shows cerebellar hypoplasia (21% reduction of perimeter) in *Chmp1a* null pups compared to control littermates. Control, n = 7; *Chmp1a* null, n = 4. (H) Quantification of (E)–(G). Two-tailed t test, *p < 0.05, **p < 0.01, ***p < 0.001. Error bars are SEM.

*Chmp1a* is required for normal embryonic development and postnatal survival in mice, with *Chmp1a* null embryos being 32% smaller than littermate controls at birth (two-tailed t test, p < 0.0001) (Figures 1A and 1C). *Chmp1a* null mice die at or soon after birth (Figure S1D), with brains that are 14% smaller than controls (two-tailed t test, p = 0.01) (Figures 1B and 1D), while *Chmp1a* heterozygous mice are indistinguishable from WT controls and hence are combined with WT in all analyses (Figures S1E and S1F). *Chmp1a* null mice have smaller olfactory bulbs, a smaller and thinner cerebral cortex, a smaller striatum, and a smaller cerebellum with reduced foliation (Figures 1E–1H and S1G). Altogether, the reduced body size, microcephaly, reduced basal ganglia, and cerebellar hypoplasia in *Chmp1a* null mice closely model the phenotype of *CHMP1A* null patients (Figure S2) (Mochida et al., 2012).

### *Chmp1a* Is Expressed in Postmitotic Neurons and Choroid Plexus during Brain Development

**Figure 2.**
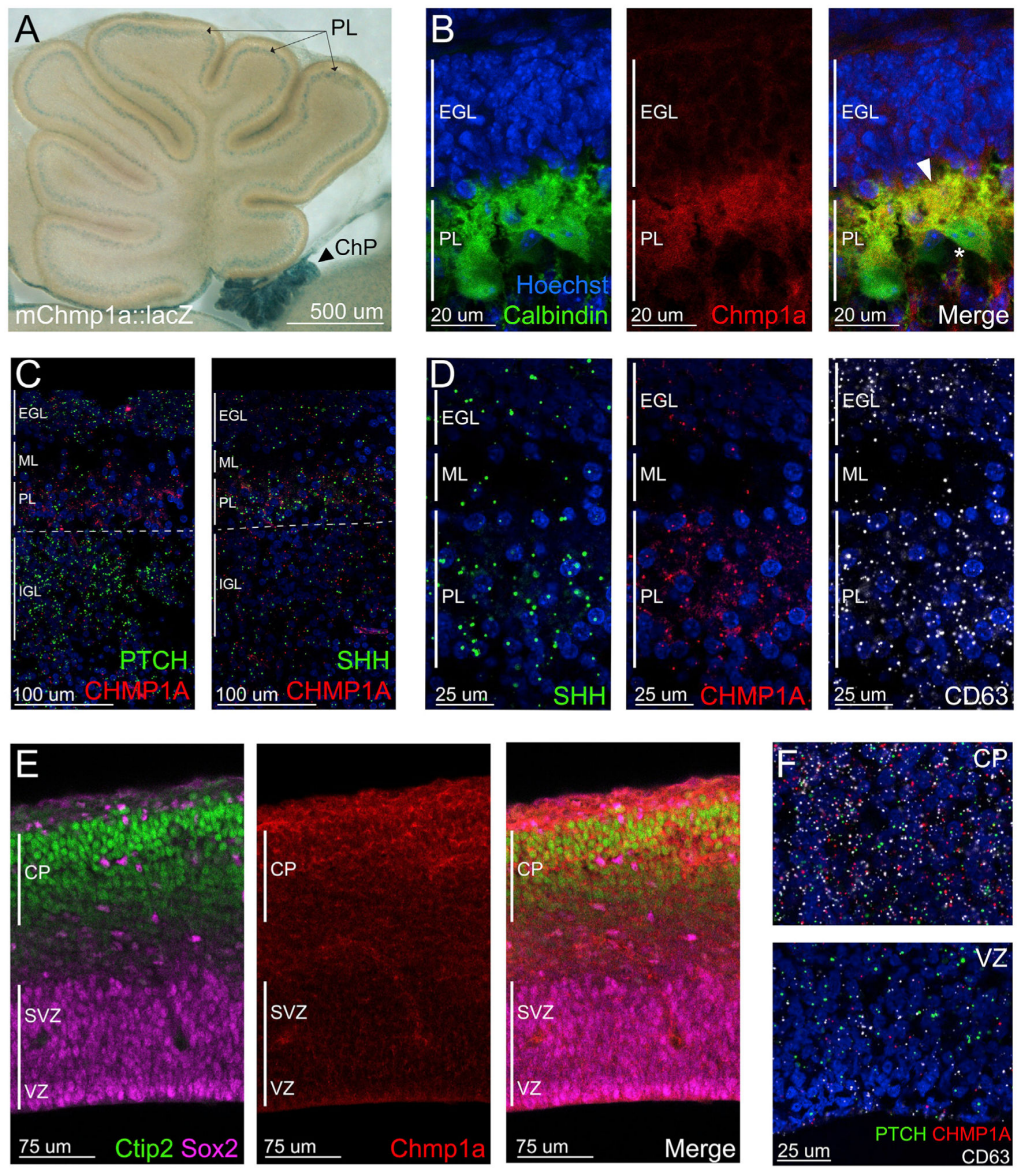
Chmp1a Is Expressed in Neurons and Choroid Plexus Epithelial Cells in Developing Brain (A) In P4 cerebellum, Chmp1a-LacZ is detected in the Purkinje cell layer and absent from EGL. LacZ is also strongly expressed in hindbrain choroid plexus (ChP, arrowhead). (B) Chmp1a immunoreactivity in cerebellar Purkinje cells (P4). Merge of Calbindin and Chmp1a highlights Chmp1a in Purkinje cell cytoplasm and dendrites (arrowhead) with absence from the nucleus (asterisk). (C) RNAscope expression of *CHMP1A*, *SHH*, and *PTCH* in developing human cerebellum. (D) RNAscope expression of *CHMP1A*, *SHH*, and *CD63* in developing human cerebellum. (E) Mouse Chmp1a protein is expressed in postmitotic neurons (Ctip2) in the cerebral cortex, but not in progenitors (Sox2) at E14.5. (F) *CHMP1A*, *PTCH*, and *CD63* expression in developing human cortex by RNAscope shows analogous localization of CHMP1A in cortical plate (CP) more than in ventricular zone (VZ). All panels are representative images of ≥2 experiments.

Insertion of lacZ into the *Chmp1a* locus provided crucial information about normal expression that suggested potential mechanisms. Heterozygous GT mice show expression of *Chmp1a-lacZ* in developing cerebellum and hindbrain choroid plexus (Figures 2A and S3C). At postnatal day (P) 4, the peak of GCP proliferation, *Chmp1a-lacZ* is specifically expressed in the PC layer (Figure 2A) and is undetectable in GCPs. Immunofluorescence confirmed the localization of Chmp1a protein in choroid plexus and in PCs in the developing cerebellum, with little expression in GCPs of the external granule layer (EGL) (Figures 2B and S3D). RNAscope in developing human cerebellum (gestational week [GW] 20) confirmed *CHMP1A* expression in the PC layer, identified as the cell layer expressing *SHH* and superficial to the *PTCH*-positive internal granule layer (Figure 2C), and further showed that *SHH*, *CHMP1A*, and CD63 are expressed in the same cell population, suggesting co-expression in PCs (Figure 2D). Immunostaining showed Chmp1a expression in AQP1-positive choroid plexus epithelial cells (Figure S3A), confirming RNA sequencing from purified epithelial cells (Lun et al., 2015). In developing mouse cerebral cortex, Chmp1a immunoreactivity is enriched in postmitotic neurons of the cortical plate compared to ventricular zone progenitors (Figure 2E), and RNAscope in developing human cortex (GW20) confirmed *CHMP1A* expression in the cortical plate, along with *CD63*, whereas the downstream *SHH* target *PTCH* is expressed in a complementary fashion in dividing progenitors of the ventricular zone (Figures 2F and S3B). The similar expression patterns of *SHH*, *CD63*, and *CHMP1A* in postmitotic neurons, with the complementary expression of *PTCH* in progenitors, suggest non-cell-autonomous roles for *CHMP1A* in proliferation via regulation of one or more secreted factors, such as SHH.

### *Chmp1a* Is Essential for Neuroprogenitor Proliferation

**Figure 3.**
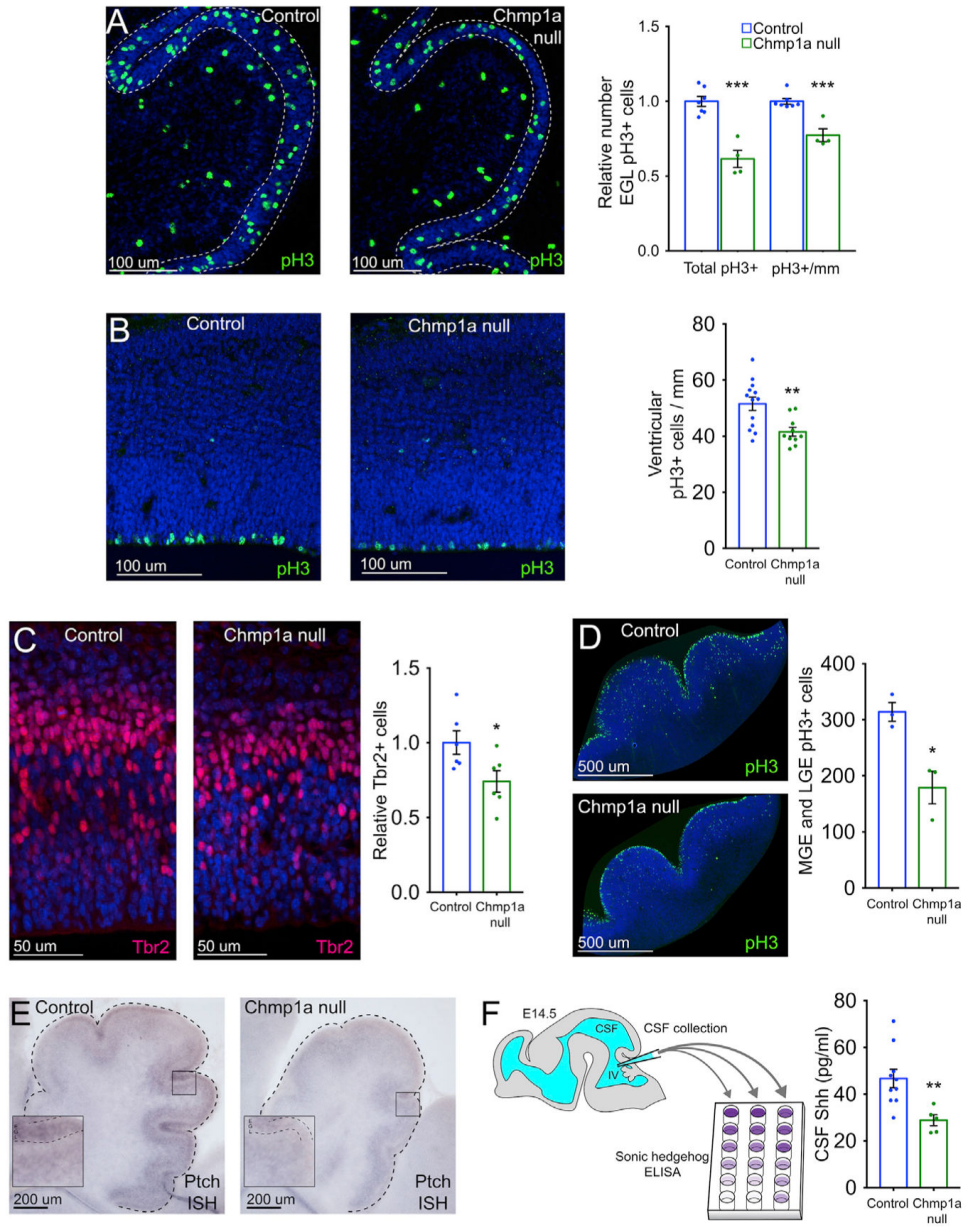
*Chmp1a* Is Required for Progenitor Proliferation in Telencephalon and Hindbrain (A) *Chmp1a* null P0 pups have 38% fewer mitotic GCPs than control littermates. Control, n = 7; *Chmp1a* null, n = 4. (B) 19% reduction in pH3-positive mitotic cortical progenitors in E14.5 *Chmp1a* null embryos. Control, n = 13; *Chmp1a* null, n = 10. (C) E13.5 *Chmp1a* null embryos have 26% fewer Tbr2-positive intermediate progenitors than controls. Control, n = 6; *Chmp1a* null, n = 6. (D) MGE and LGE show 43% fewer pH3-positive mitotic progenitors in *Chmp1a* null embryos. Control, n = 3; *Chmp1a* null, n = 3. (E) *In situ* hybridization (ISH) for *Ptch* shows reduced expression in *Chmp1a* null P0 cerebellum. Control, n = 8; *Chmp1a* null, n = 3. (F) CSF concentration of Shh in the 4^th^ ventricle at E14.5 is reduced 38% in *Chmp1a* null embryos. Control, n = 10; *Chmp1a* null, n = 5. Two-tailed, unpaired t test, *p < 0.05, **p < 0.01. Error bars are SEM.

*Chmp1a* null mouse embryos show defects in progenitor proliferation in cerebellum, cortex, and basal ganglia (Figure 3). At P0, the latest age we can study due to lethality, GCP proliferation in the developing cerebellum is significantly impaired in *Chmp1a* null mice with 38% fewer mitotic GCPs than littermate controls (labeled with phosphorylated histone H3 [pH3], two-tailed t test, p = 0.0001) (Figure 3A). This deficit is twice as large as the reduction in mitotic progenitors in the developing cortex and matches human *CHMP1A* null patients, whose cerebellar hypoplasia is strikingly severe in relation to more modest microcephaly (Figure S2) (Mochida et al., 2012). The overall size of the cerebellum is already reduced at P0 in *Chmp1a* null mice (Figure 1G and 1H), with a 23% reduction in mitotic GCP density compared to controls (two-tailed t test, p = 0.0003) (Figure 3A), whereas PCs are not detectably affected in mutant mice (Calbindin-positive area: 37% versus 37%, two-tailed t test, p = 0.97) (Figures S4A and S4B).

*Chmp1a* null embryos have decreased anterior-to-posterior cerebral cortex length (Figure 1B), a 13% thinner cortex (two-tailed t test, p = 0.03), and defects in cortical layers (Figures 1E and 1H), all suggesting defects in cortical neurogenesis. Superficial cortical layers (II-IV, Cux1-positive neurons) are reduced by 25% in the mutants (two-tailed t test, p = 0.002), while deep cortical layers (V-VI, Ctip2-positive neurons) are less affected (9% reduction, two-tailed t test, p = 0.08) (Figures 1E and 1H). Preferential reduction of upper cortical layers commonly reflects defects of cortical neurogenesis (Lizarraga et al., 2010), because cortical layers form in an inside-out sequence, with deep-layer neurons born first and upper-layer neurons born last (Custo Greig et al., 2013). Quantification of mitotic ventricular zone progenitors at embryonic day (E) 14.5 showed 19% fewer mitotic ventricular zone progenitors in mutants compared to controls (two-tailed t test, p = 0.004) (Figure 3B), and at E13.5, mutant embryos had 26% fewer Tbr2-positive intermediate progenitors than controls (two-tailed t test, p = 0.035) (Figure 3C). *Chmp1a* null embryos had no detectable increase in cleaved caspase-3-positive apoptotic cells (Figures S4C and S4D) and intact epithelial structure with normal localization of key proteins such as atypical protein kinase C (aPKC) and β-catenin (Figure S4E), suggesting that the microcephaly and cortical thinning result from decreased progenitor proliferation. Mutant embryos also showed normal progression of cytokinesis in cortical progenitors measured by the ratio of pH3-positive early mitotic cells to aurora A-positive cells undergoing abscission (Figures S5A and S5B) and normal DNA content measured by propidium iodide staining of mouse embryonic fibroblasts (MEFs) (Figure S5D). These data suggest loss of *Chmp1a* causes defects in proliferation, but not cytokinesis, a process in which the ESCRT complex has also been implicated (Carlton et al., 2012).

In late-embryonic ventral telencephalon, the striatum is 25% smaller in the absence of *Chmp1a* (two-tailed t test, p = 0.0004) (Figures 1F and 1H), which results from decreased progenitor proliferation at E12.5 in medial ganglionic eminence (MGE) and lateral ganglionic eminence (LGE). Immunostaining for pH3 during MGE and LGE neurogenesis (E12.5) shows a dramatic 43% reduction in pH3-positive progenitors in *Chmp1a* null embryos (two-tailed t test, p = 0.016) (Figure 3D), revealing essential roles for *Chmp1a* in ventral telencephalon that are also seen in humans with *CHMP1A* mutations (Figure S2).

### *Chmp1a* Is Essential for Shh-Mediated Progenitor Proliferation

Because Shh is the primary mitogen that drives GCP proliferation (Corrales et al., 2004; Dahmane and Ruiz i Altaba, 1999), reduced GCP proliferation in *Chmp1a* null mice prompted examination of whether *Chmp1a* is required for Shh-mediated proliferation. In control mice at birth, *Shh* signaling is more active anteriorly than dorsally in the cerebellum (Corrales et al., 2004), with almost twice the number of mitotic GCPs (pH3+) in anterior EGL compared to dorsal EGL. In *Chmp1a* null mice, this difference was substantially reduced to 1.28-fold (two-tailed t test, p = 0.005) (Figures S4F and S4G), consistent with defective *Shh* signaling. *In situ* hybridization for *Ptch*, a downstream target of *Shh* signaling, revealed lower *Ptch* expression in *Chmp1a* null cerebellum that was especially pronounced in the Shh-responsive EGL (Figure 3E), providing further evidence for decreased *Shh* signaling in the absence of *Chmp1a*.

### *Chmp1a* Is Required for Shh Secretion *In Vivo*

Direct measurement of total Shh protein concentration with a highly sensitive ELISA showed 38% less Shh in embryonic CSF from *Chmp1a* null mouse embryos compared to controls at E14.5 (two-tailed t test, p = 0.009) (Figure 3F), providing direct evidence that *Chmp1a* is required for Shh secretion in the developing brain. In parallel, smoothened agonist (SAG) stimulation of *Chmp1a* null and control MEFs showed no significant defect in downstream *Shh* signaling, as measured by *Gli1* activation (two-tailed t test, p = 0.39) (Figure S5H), consistent with *Chmp1a* acting upstream of *Shh*. Furthermore, we performed a rescue experiment with a *Ptch* heterozygous null mouse, a genetic tool that increases *Shh* signaling *in vivo* (Goodrich et al., 1997), and found that *Chmp1a* null embryo microcephaly at E18.5 was reversed in *Chmp1a* null:*Ptch* heterozygous embryos and not significantly different from control (two-tailed t test, p = 0.22) (Figure S5G). This result provides additional evidence that microcephaly in *Chmp1a* null mice reflects decreased *Shh* function.

### *CHMP1A* Is Essential for Human Cerebral Organoid Formation

**Figure 4.**
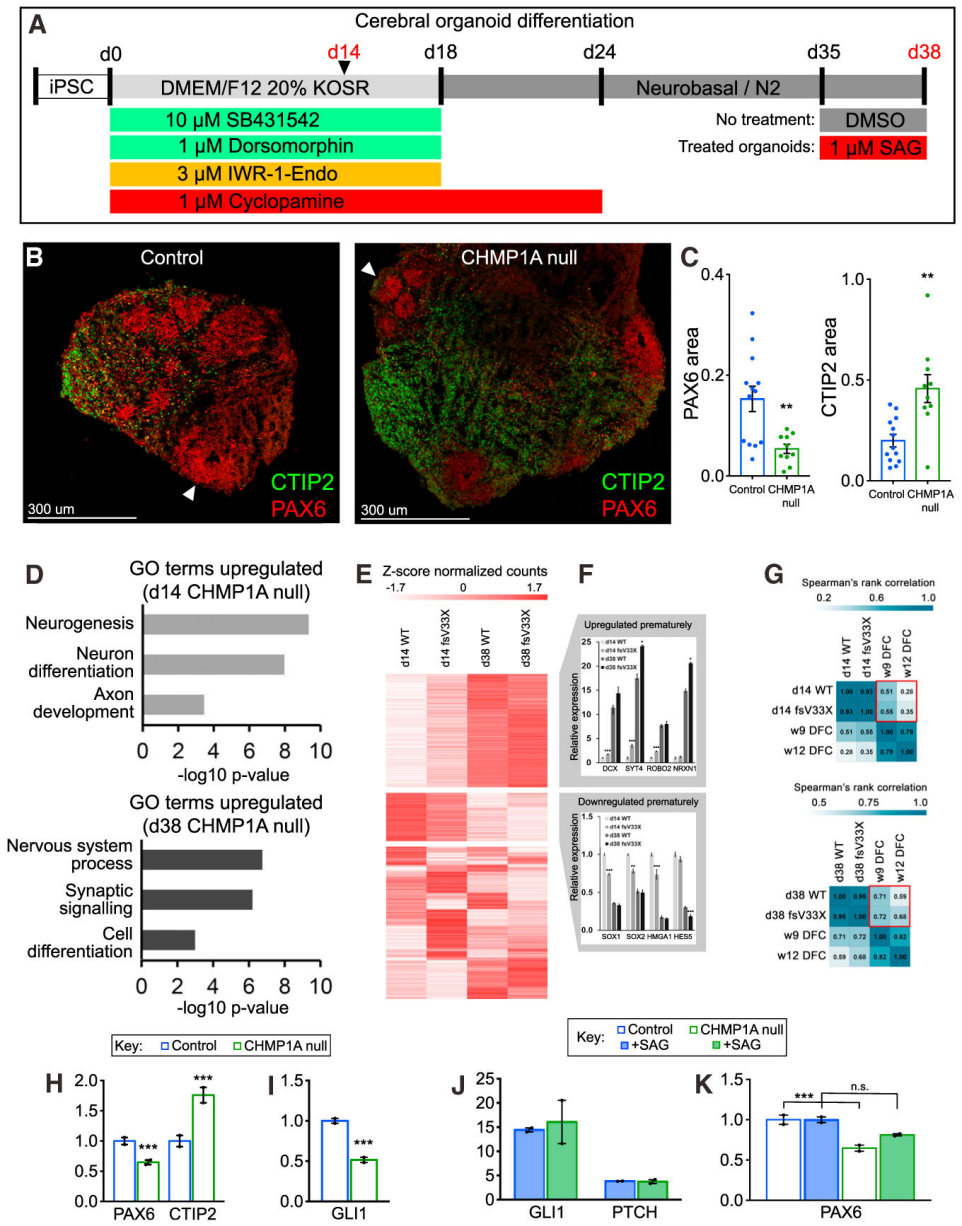
*CHMP1A* Is Essential for Progenitor Maintenance in Human Cerebral Organoids (A) Human cerebral organoid differentiation protocol. (B) Organoids contain cortical progenitors (red, immunostained for PAX6, arrowheads) surrounded by postmitotic neurons (green, immunostained for CTIP2). (C) PAX6 area is decreased and CTIP2 area is increased in *CHMP1A* null organoids. Control, n = 13; *Chmp1a* null, n = 10. (D) GO pathway enrichment analysis. (E) RNA sequencing reveals clusters of genes up-and downregulated in *CHMP1A* null organoids. (F) Expression of differentiation and postmitotic neuron markers is increased in *CHMP1A* null organoids, whereas expression of proliferative markers is decreased. (G) Comparison of organoid gene expression profiles to expression profiles of human fetal cortex. (H) Decreased *PAX6* expression and increased *CTIP2* expression by RNA sequencing at day 38 in *CHMP1A* null organoids. (I) Decreased *GLI1* expression in *CHMP1A* null iPSCs. (J) SAG treatment of organoids induces *GLI1* and *PTCH* expression. (K) SAG treatment partially rescues decreased *PAX6* expression in *CHMP1A* null organoids. (C and J) Two-tailed, unpaired t test. (F, H, I, and K) DESeq2 adjusted p value (Wald test). *p < 0.05, **p < 0.01, ***p < 0.001. Error bars are SEM.

To further examine *CHMP1A*’s role in SHH-driven neuroprogenitor proliferation, we generated *CHMP1A* null human cerebral organoids using CRISPR/Cas9 mutagenesis and found impaired progenitor maintenance and premature neuronal differentiation (Figures 4A and S6A). Immunostaining of day-38 organoids for PAX6 and CTIP2 showed a 65% decrease of PAX6-positive progenitor cell area and 130% increase of CTIP2-positive postmitotic neuron area (two-tailed t test; CTIP2, p = 0.001; PAX6, p = 0.003) (Figures 4B and 4C). RNA sequencing of induced pluripotent stem cells (iPSCs) before differentiation, organoids at day 14 during differentiation, and organoids at day 38 after differentiation (Figures S6B and S6C) further defined the defect in organoid formation as impaired progenitor maintenance and premature neuronal differentiation. Gene ontology (GO) pathway analysis of upregulated genes in *CHMP1A* null organoids showed enrichment of terms for neuronal differentiation (Figure 4D). RNA sequencing revealed distinct groups of up- and downregulated genes in the absence of *CHMP1A*, including loss of progenitor markers *SOX1*, *SOX2*, *HMGA1*, and *HES5*) early induction of differentiated neuron markers (*DCX*, *SYT4*, *ROBO2*, and *NRXN1*) (DESeq2 adjusted p value, each gene: p < 0.05) (Figures 4E and 4F; Tables S1 and S2). *CHMP1A* null day-38 organoids correlated more closely with RNA sequencing data from GW12 human cortex (Allen Institute), while control organoids correlated more closely with GW9 cortex, providing additional evidence for premature differentiation (Figure 4G). By RNA sequencing at day 38, *CHMP1A* null organoids show decreased expression of progenitor marker *PAX6* and increased expression of postmitotic neuron marker *CTIP2* (Figure 4H).

RNA sequencing revealed impaired *SHH* signaling with 48% reduction of *GLI1* expression in mutant iPSCs at the start of organoid differentiation (DESeq2 adjusted p value, iPSC: p < 0.0001) (Figure 4I). Activation of *SHH* signaling with exogenous administration of SAG on differentiation day 35 (with harvest on day 38) (Figure 4A) induced *GLI1* and *PTCH* expression equally in WT and *CHMP1A* null organoids (two-tailed t test; *GLI1*, p = 0.75; *PTCH*, p = 0.84), suggesting no downstream *SHH* signaling defect in the absence of *CHMP1A* (Figure 4J). Rather, *SHH* signaling activation rescued decreased expression of *PAX6* in *CHMP1A* null organoids, a defining marker of impaired progenitor maintenance in the absence of *CHMP1A* (DESeq2 adjusted p value; *PAX6* no treatment, p < 0.0001; *PAX6* +SAG, p = 0.11) (Figure 4K), although some gene expression differences were not rescued (Figure S7). These data suggest that progenitor proliferation and maintenance defects in the absence of *CHMP1A* result from upstream impairment of *SHH* signaling via decreased SHH secretion, although other functions of *CHMP1A* may also play a role.

### Defective ILV and MVB Structure in *Chmp1a* Null Embryos

**Figure 5.**
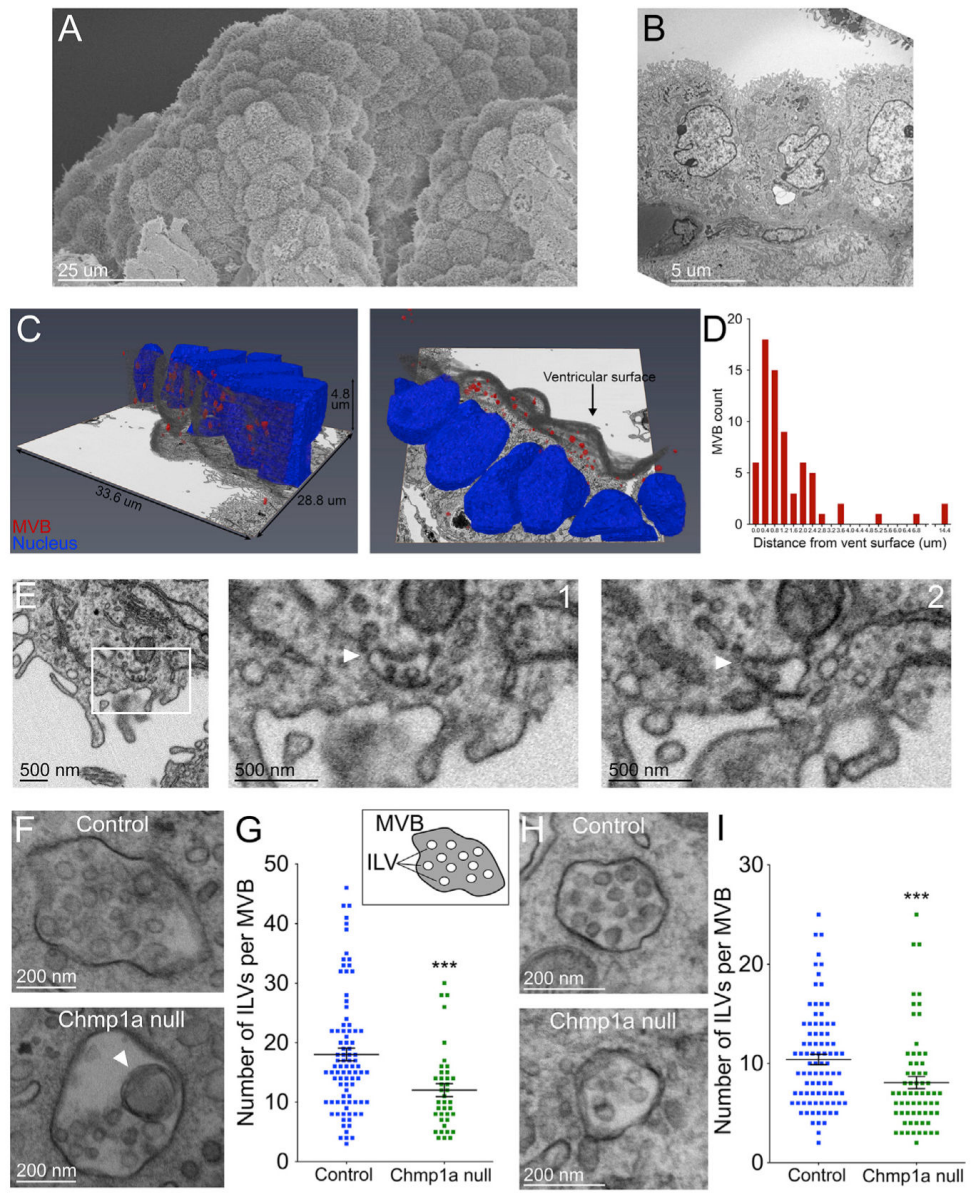
*Chmp1a* Is Essential for ILV Formation in Developing Mouse Brain (A) SEM of mouse embryonic hindbrain choroid plexus. (B) TEM of mouse embryonic hindbrain choroid plexus. (C) FIB-SEM of mouse choroid plexus revealed accumulation of MVBs near the epithelial cell ventricular surface. (D) Quantification of MVB distribution within epithelial cells. (E) MVB fused with the plasma membrane of the choroid plexus epithelial cell (arrowhead). Zoom shows two sequential z slices. (F)Fewer ILVs in *Chmp1a* null choroid plexus MVBs and abnormally large ILVs (arrowhead). (G) Quantification of (F). Control, n = 88 MVBs (7 embryos); *Chmp1a* null, n = 39 MVBs (2 embryos). (H) Fewer ILVs in *Chmp1a* null Purkinje cells. (I) Quantification of (H). Control, n = 88 MVBs (6 embryos); *Chmp1a* null, n = 65 MVBs (2 embryos). (G and I) Mann-Whitney test, ***p < 0.001. Error bars are SEM.

Because Shh secretion has been reported on EVs and ESCRT-III is involved in ILV biogenesis in MVBs, we used three-dimensional focused ion beam (FIB) scanning electron microscopy (SEM) (Xu et al., 2017) to examine choroid plexus (ChP) epithelial cells, a primary source of Shh in embryonic mouse brain (Nielsen and Dymecki, 2010; Lun et al., 2015), and found abundant MVBs near the ventricular surface. SEM revealed choroid plexus’s remarkable structure as a monolayer of epithelial cells forming grape-like clusters that maximize ventricular surface area (Figure 5A) and transmission electron microscopy (TEM) showed the ventricular surface cross-section (Figure 5B). FIB-SEM imaging revealed that 75% of MVBs in choroid plexus epithelial cells were located within 2 μm of the ventricular surface, raising the possibility that some could be secretory MVBs (Figures 5C and 5D). Supporting this idea, one MVB was in contact with the ventricular surface membrane (Figure 5E). CHMP1A immunoreactivity in human hindbrain choroid plexus epithelial cells distributed with CD63-immunoreactive MVBs near the ventricular surface (Figure S8A), providing additional evidence of secretory MVBs. MVB fusion with the plasma membrane releases ILVs as EVs and exosomes (Tietje et al., 2014). Thus, our imaging provides evidence that choroid plexus epithelial cells are a source of EVs in the developing brain.

TEM showed disrupted MVB structure in *Chmp1a* null embryonic choroid plexus. The number of ILVs per MVB was 37% lower in mutant choroid plexus epithelial cells than in control (Mann-Whitney test, p = 0.0003) (Figures 5F, 5G, and S8C), and some *Chmp1a* null MVBs contained abnormally large ILVs (Figure 5F, arrowhead), a known consequence of impaired ILV budding (Lee et al., 2007). Apart from the observed defects in EV biogenesis, the choroid plexus ventricular surface and microvilli appeared otherwise normal in *Chmp1a* null embryos (Figure S8B). As in choroid plexus epithelial cells, P0 mutant cerebellar PC MVBs showed 24% fewer ILVs per MVB compared to controls (Mann-Whitney test, p = 0.0005) (Figures 5H, 5I, and S8D). These data raise the possibility that defective Shh secretion from choroid plexus in CSF and from PCs in cerebellum may involve defective ILV formation in the MVB. In addition, we did not observe defects in two other ESCRT functions, cytokinesis and epidermal growth factor receptor (EGFR) degradation, in the absence of *Chmp1a*. As described earlier, cytokinesis was not impaired in *Chmp1a* null cortical progenitors and *CHMP1A* null iPSCs (Figures S5A–S5C). We measured degradation of EGFR following EGF stimulation—an established measure of ESCRT-mediated MVB-to-lysosome maturation (Slagsvold et al., 2006)—in HeLa cells and found no detectable difference in EGFR degradation in *CHMP1A*-depleted cells compared to controls (Figures S5E and S5F).

TEM showed MVBs are abundant in dendrites of both PC and cortical projection neurons. At P4, cerebellar PC MVBs are common in the dendrites that extend toward the EGL, where Shh-responsive GCPs are located (Figure S9A). In mouse cerebral cortex, serial reconstruction of hundreds of TEM images of cortical pyramidal neurons (Lee et al., 2016) shows that MVBs are surprisingly abundant in pyramidal cell dendrites (Figure S9B). The dendritic arbor and axon of a single pyramidal cell contained at least 80 MVBs, often near synapses, which could be interpreted as active dendritic EV secretion sites. Shh released from pyramidal cell dendrites during postnatal development is required for synapse formation between layer V corticofugal projection neurons and callosal projection neurons in the cortex (Harwell et al., 2012). These findings suggest EV-mediated secretion is widespread across multiple time points and anatomic regions of the developing brain.

### *CHMP1A* Is Required for Vesicular SHH Secretion *In Vitro*

**Figure 6.**
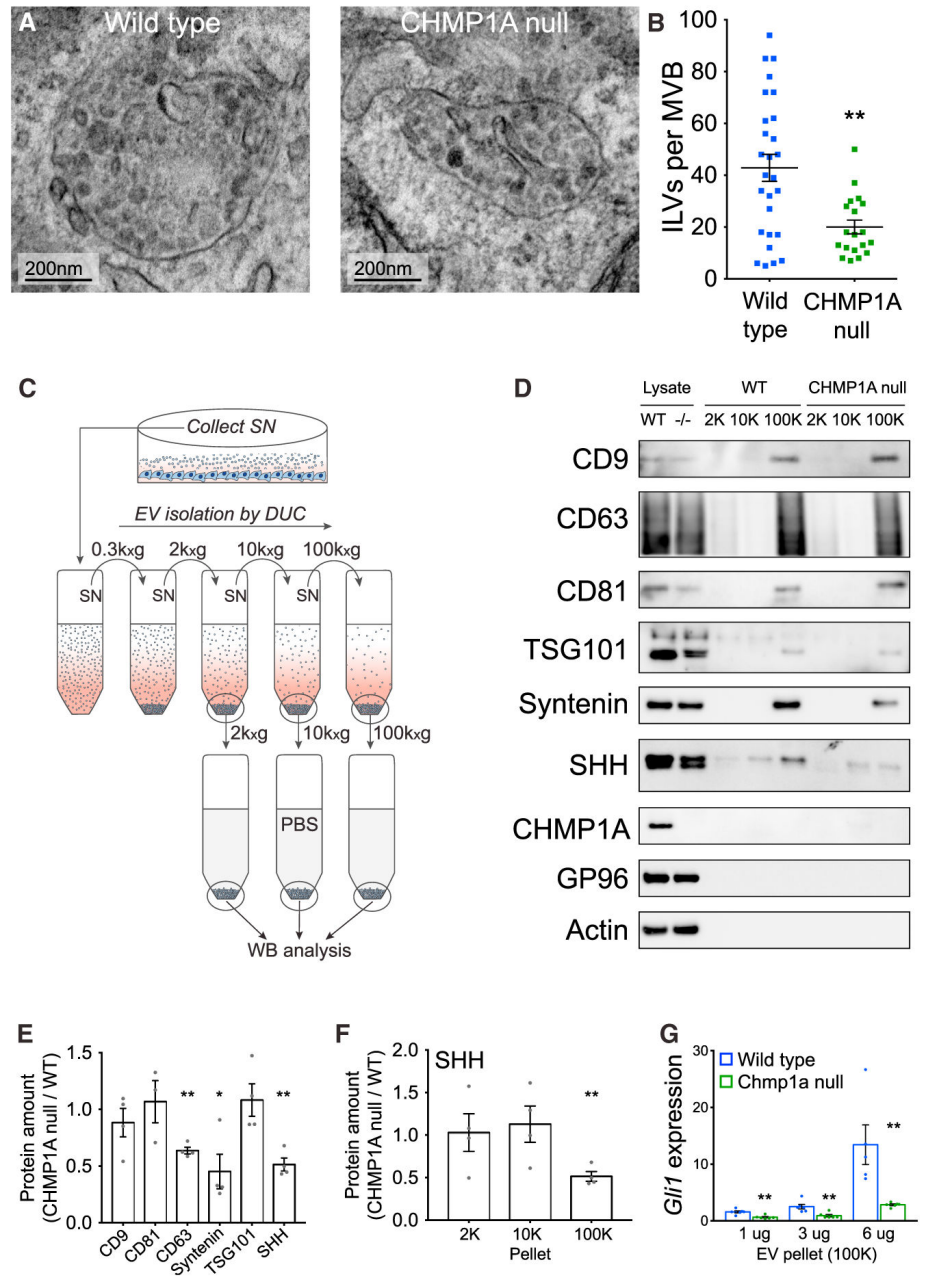
*CHMP1A* Depletion Decreases Secretion of SHH and Exosomes *In Vitro* (A) Decreased ILV formation in MVBs of *CHMP1A* null SVG-A cells compared to WT (TEM). (B) Quantification of (A). Wild-type, n = 27; *CHMP1A* null, n = 19. (C) EV isolation procedure by differential ultracentrifugation (DUC) (Kowal et al., 2016). (D) Representative WB analysis of isolated EVs (2K, 10K, and 100K pellets) from WT and *CHMP1A* null SVG-A cells expressing SHH. Blot shows EV-specific markers (CD9, CD63, CD81, Syntenin, and TSG101), EV-excluded markers (GP96 and Actin), CHMP1A, and SHH. (E) Quantification of (D). Wild-type, n = 4; *CHMP1A* null, n = 4. (F) Quantification of SHH WB signals in the 2K, 10K, and 100K fractions. Wild-type, n = 4; *CHMP1A* null, n = 4. (G) *Gli1* induction in NIH 3T3 cells induced by 100K pellet from SHH-transfected SVG-A cells. 1 and 3 μg: wild-type, n = 6; *CHMP1A* null, n = 6. 6 μg: wild-type, n = 5; *CHMP1A* null, n = 6. (B) Mann-Whitney test. (E–G) Two-tailed, unpaired t test. *p < 0.05, **p < 0.01. Error bars are SEM.

To dissect the mechanism underlying regulation of SHH secretion by *CHMP1A*, we used the human fetal glial cell line SVG-A and generated *CHMP1A* null monoclonal lines through CRISPR/Cas9 mutagenesis (Figures S10A and S10B). TEM of mutant cells showed a 57% reduction of ILVs per MVB in *CHMP1A* null cells compared to WT (Mann-Whitney test, p = 0.005) (Figures 6A and 6B), confirming our *in vivo* results from choroid plexus and PCs (Figure 5). Serial ultracentrifugation of conditioned medium obtained from *SHH*-expressing SVG-A cells enabled EV collection and initial separation based on size (Figure 6C) (Experimental Procedures) (Kowal et al., 2016): large EVs and cell debris pelleted in the 2,000 × *g* (2K) fraction, medium-sized EVs pelleted in the 10,000 × *g* (10K) fraction, and small EVs, including microvesicles and exosomes, pelleted in the 100,000 × *g* (100K) fraction. The exosome-enriched markers CD9, CD81, CD63, TSG101, and Syntenin were either restricted to or highly concentrated in the 100K pellet, while exosome-excluded markers, such as actin and endoplasmic reticulum (ER)-resident protein GP96 were absent from this fraction (Figure 6D), confirming previous reports (Lötvall et al., 2014). In the *CHMP1A* null 100K pellet, CD63 was decreased 36% and Syntenin was decreased 55% (two-tailed t test; CD63, p = 0.001; Syntenin, p = 0.037), while CD9, CD81, and TSG101 were not detectably changed (two-tailed t test; CD9, p = 0.42; CD81, p = 0.75; TSG101, p = 0.61) (Figure 6E). Although SHH was detected in all vesicular fractions, it was significantly decreased only in the 100K EV fraction derived from *CHMP1A* null cells (49% reduction, two-tailed t test, p = 0.003) (Figure 6F). Similar results were obtained using a heterogeneous pool of *CHMP1A* null cells (Figure S10C) instead of a monoclonal population. The 100K fraction contained biologically active SHH that induced *Gli1* expression in NIH 3T3 cells. At equal protein content, the signaling potency of the 100K EV fraction from *CHMP1A* null cells was reduced 79% compared to EVs of WT cells (6 μg protein, two-tailed t test, p = 0.008) (Figure 6G), demonstrating that *CHMP1A* is required for secretion of active SHH-containing EVs.

### SHH Is Secreted on a Specific EV Subtype

**Figure 7.**
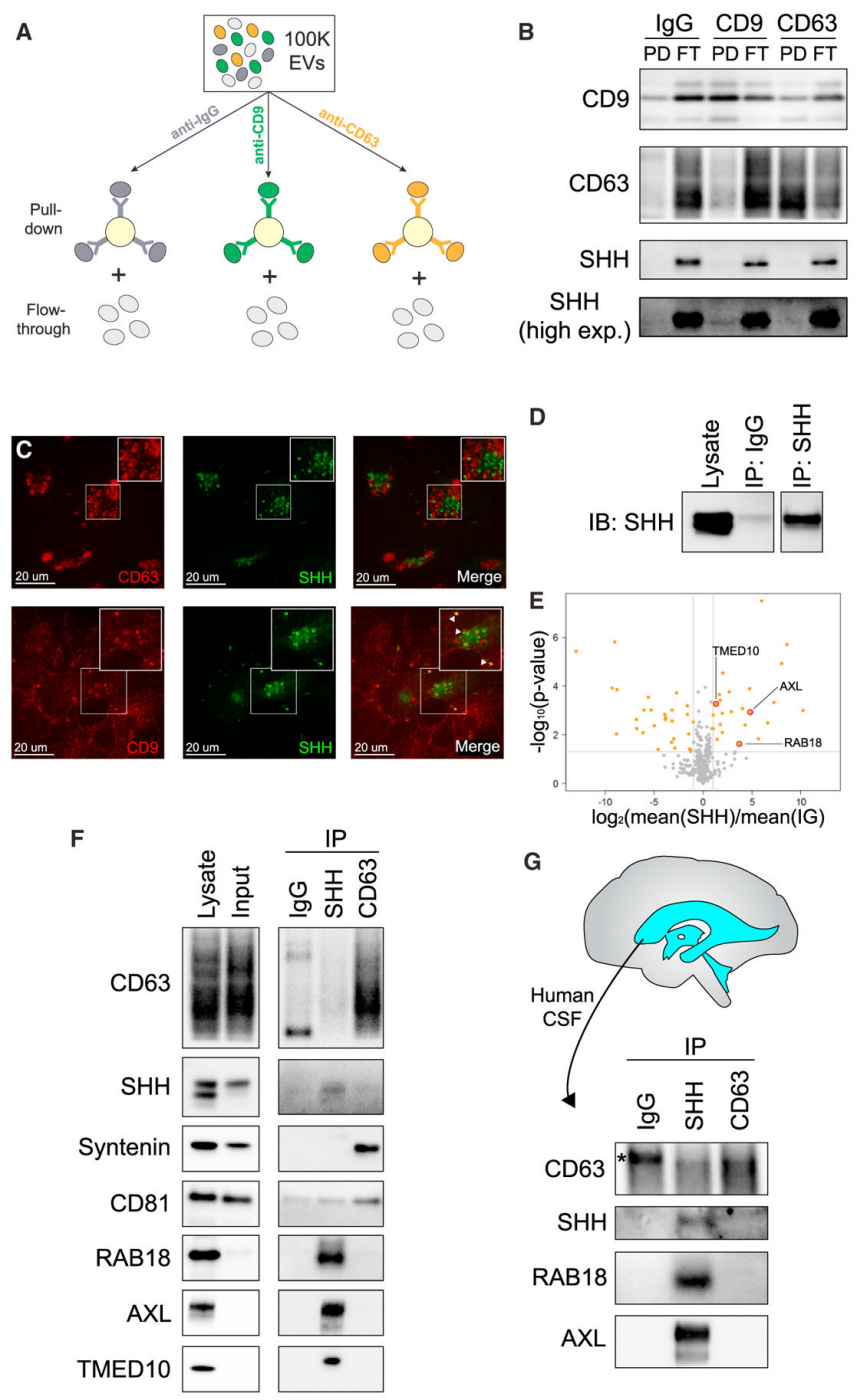
SHH Is Secreted on a Specific EV Subtype, ART-EV (A)Scheme of exosome isolation from the 100Kpellet by immunoprecipitation using beads coupled to CD9 and CD63 antibodies or mouse pan-immunoglobulin G (IgG) antibodies. Both bound (pull-down) and unbound (flow-through) material was recovered. (B) Representative WB of the bound (PD) and unbound (FT) fractions from exosome immunoisolation, probed with antibodies against CD9, CD63, and SHH (n = 3). Most SHH does not co-purify with exosomes but remains in the FT fraction. (C) Immunostaining of exosomal markers CD9 or CD63 in cells ectopically expressing mNeonGreen-SHH. (D) SHH-positive vesicles were purified from the 100K pellet by immunoisolation using anti-SHH antibodies. (E) Peptides enriched in EVs isolated by SHH immunoisolation. (F) Immunoisolated SHH-positive vesicles and CD63-positive exosomes were subjected to WB analysis, confirming the exclusive presence of RAB18, AXL, and TMED10 in purified SHH vesicles (n = 3). In contrast, Syntenin is only found on CD63-positive exosomes, while CD81 is present on both types of vesicles. (G) SHH- and CD63-positive vesicles from the 100K pellet were immunoisolated from human CSF and subjected to WB analysis, confirming the exclusive presence of RAB18 and AXL on ART-EVs *in vivo*. * indicates non-specific band.

Because the 100K pellet formed following ultracentrifugation is a complex mixture of several distinct EV subtypes, we used immunoisolation to determine which subtype was SHH positive (Figure 7A) (Kowal et al., 2016). We immunoisolated with anti-CD9 or anti-CD63 antibodies to test for the presence of SHH on exosomes. Western blot (WB) analysis of bound and unbound material showed modest SHH amounts on CD9-positive EVs and minimal SHH on CD63-positive EVs (Figure 7B). Similarly, we observed minimal co-localization of SHH and either CD9 or CD63 by confocal immunofluorescence microscopy (Figure 7C), suggesting that most SHH in the 100K fraction is secreted on vesicles distinct from classical exosomes.

To define the hallmark of SHH-bound vesicles, we immunoisolated SHH-positive EVs from the 100K pellet and subjected the bound material to mass spectrometry analysis to identify other components of these EVs (Figure 7D). This analysis revealed a unique set of proteins significantly enriched in the SHH fraction, including Ras-related protein Rab-18 (RAB18), tyrosine-protein kinase receptor UFO (AXL), and transmembrane emp24 domain-containing protein 10 (TMED10), that have not been reported as major components of exosomes (Welch’s t test; RAB18, p = 0.008; AXL, p < 0.001; TMED10, p = 0.001) (Figure 7E; Table S3) (Lötvall et al., 2014). Moreover, the exosomal markers CD63, CD9, and Syntenin were not enriched, while CD81 was present (Table S3). By immunoprecipitation of CD63 or SHH, we found that RAB18, AXL, and TMED10 mark a specific EV subtype that is distinct from CD63-positive exosomes (Figure 7F), which we label ART-EV (AXL, RAB18, and TMED10 EV).

### CHMP1A Is Present on ART-EVs

ESCRT components participate in the process of exosome biogenesis, but they are also found associated with EVs in the extracellular environment (Chiasserini et al., 2014). To investigate whether CHMP1A is associated with ART-EVs, we isolated SHH-containing vesicles from the 100K fractions of SVG-A WT and *CHMP1A* null cells by immunoprecipitation. WB analysis of the bound fractions revealed the presence of CHMP1A only in ART-EVs recovered from WT cells, confirming the specificity of the detection (Figure S10D). In contrast, AXL and RAB18 were similarly detected in ART-EVs from both WT and *CHMP1A* null cells. High-resolution time-lapse confocal microscopy of the intracellular co-distribution of ectopically expressed SHH and CHMP1A in live SVG-A cells showed that both proteins distributed mostly in independent compartments, but a subset of CHMP1A and SHH structures overlap over time (Figure S10E; Video S1). Altogether, these findings suggest that CHMP1A physically associates with SHH-positive ART-EVs. To confirm that ART-EVs are present in the adult nervous system *in vivo*, we isolated the 100K EV fraction from human adult CSF (collected from a patient with glioblastoma multiforme) and purified SHH-containing vesicles, as well as CD63-positive exosomes from the 100K CSF pellet, by immunoprecipitation. Pulldown of SHH co-purified AXL and RAB18, which were absent from the CD63-positive exosome fraction (Figure 7G), thus confirming the specific protein signature of ART-EVs identified in SVG-A cells. Altogether, our data strongly suggest that SHH is secreted *in vivo* on a distinctive EV subtype: ART-EV.

## DISCUSSION

By characterizing *Chmp1a* null mice and *CHMP1A* null human cerebral organoids, we show that *CHMP1A* is essential for neural progenitor proliferation and maintenance. Furthermore, we demonstrate that ESCRT-mediated release of EVs is impaired in the absence of *CHMP1A* and that this reduces secretion of vesicle-bound SHH, a key driver of progenitor proliferation in the brain. The ESCRT complex has many cellular functions, and loss of an ESCRT-III component could cause numerous defects; however, we show that loss of *CHMP1A* is relatively specific, decreasing the number of ILVs within the MVB, but not impairing cytokinesis or EGFR degradation. We show *CHMP1A* is required for EV secretion, and although this defect could impair multiple developmental pathways, essentially all neurodevelopmental defects seen in *Chmp1a* null mice (and *CHMP1A*-deficient humans) can be ascribed to impaired *SHH* signaling and the specific defect in SHH secretion we demonstrate. We define a specific EV subtype, ART-EV, on which SHH is secreted, and show that ART-EVs exist *in vivo*. Our findings provide evidence for a previously undescribed mechanism of SHH secretion in vertebrate brain development with broad potential relevance to secretion of growth factors and bioactive molecules in the CNS.

### *Chmp1a* Null Phenotype Is Consistent with Hypomorphic *Shh* Signaling

Several of the most obvious neurodevelopmental defects in *Chmp1a* null mice—including a thin cerebral cortex, small basal ganglia, cerebellar hypoplasia, small somatic size, and perinatal lethality—are consistent with partial loss of *Shh*, because these defects have been observed in previous mouse models of decreased *Shh* signaling through hypomorphic alleles (Huang et al., 2007; Chamberlain et al., 2008). In addition, both impaired progenitor maintenance in *CHMP1A* null organoids and microcephaly in *Chmp1a* null mice can be partially rescued by downstream *Shh* signaling activation either chemically or genetically. However, the lack of digit or spinal cord patterning defects in *Chmp1a* null mice compared to null *Shh* mutations (Zhu and Mackem, 2011; Fuccillo et al., 2006) is consistent with our observed incomplete blockage of Shh secretion and the observation that spinal cord Shh is secreted on large EVs (Tanaka et al., 2005), whereas depletion of *CHMP1A* specifically impairs secretion of SHH on small EVs (Figure 6F). However, the absence of these phenotypes and the lack of strong dysregulation of *SHH* signaling in *CHMP1A* null organoids, as well as incomplete correction of defects in some progenitor and differentiation markers in *CHMP1A* null organoids by SAG rescue (Figure S7), highlights the complexity of the system and leaves open the possibility that *CHMP1A* may regulate other pathways.

### Multiple Specialized SHH Secretion Mechanisms

*In vivo* Hedgehog (Hh) secretion has been studied primarily in the *Drosophila* imaginal disk, where the proposed underlying mechanism remains controversial. Matusek et al. (2014) showed Hh secretion occurs on larger EVs called ectosomes released from the plasma membrane in an ESCRT-dependent manner. In their study, disruption of MVB biogenesis did not impair Hh secretion, suggesting it did not occur via MVB-derived exosomes. In contrast, Parchure et al. (2015) reported Hh secretion via ESCRT-mediated exosome-like vesicles derived from MVBs. Shh secretion at the embryonic mouse ventral node appears to occur via large, plasma membrane-derived EVs (Tanaka et al., 2005), which are not obviously impaired in the absence of *Chmp1a*. These results highlight the complexity and confusion surrounding vesicular Shh secretion. Our finding that small EVs distinct from exosomes carry Shh in developing mammalian brain highlights the diverse mechanisms of Shh secretion.

### SHH Secretion via ART-EVs

We report SHH secretion on a specific EV subtype, ART-EV, defined by marker proteins that distinguish it from CD63-positive exosomes. To our knowledge, RAB18, AXL, and TMED10 have not been previously reported as components of SHH-positive EVs and thus can provide insights into the mechanism of SHH secretion. RAB18 is a protein with emerging roles in ER to Golgi retrograde trafficking and other cellular trafficking (Vazquez-Martinez et al., 2007; Dejgaard et al., 2008), and LOF mutations in *RAB18* are associated with Warburg micro syndrome, establishing a critical role of *RAB18* in eye and brain development and neurodegeneration (Bem et al., 2011). The transmembrane proteins AXL and TMED10 may function in biogenesis of specific vesicles in SHH-producing cells, because AXL is a receptor tyrosine kinase expressed at the plasma membrane that is required in microglia for apoptotic cell removal (O’Bryan et al., 1991; Fourgeaud et al., 2016) and TMED10 (TMP21/P23) is a transmembrane protein that is involved in early secretory pathway trafficking and is a member of the Presenilin complex (Blum et al., 1996; Chen et al., 2006).

### *CHMP1A* Regulates Secretion of Multiple EV Subtypes

Previous studies have established important roles for different members of the ESCRT machinery in ILV biogenesis (Colombo et al., 2013; Baietti et al., 2012). We found fewer ILVs within MVBs in the absence of *CHMP1A* and, consistent with this phenotype, observed a decrease in secretion of CD63-positive EVs. Exosomes have been defined as EVs of MVB origin co-enriched in tetraspanins CD9, CD81, and CD63 and endosome markers TSG101 or Syntenin (Lötvall et al., 2014). However, in our experimental system, we observed reduced secretion of some exosome markers (CD63 and Syntenin), but not others (CD9, CD81, and TSG101), indicating the existence of a heterogeneous EV population in the 100K fraction. This finding supports previous work suggesting that MVB-derived EVs are heterogeneous with respect to their size and cargo, that cells contain distinct MVBs, and that ILVs of the same MVB display different structures and compositions (Colombo et al., 2013; Bobrie et al., 2012; van Niel et al., 2001; Février and Raposo, 2004; Möbius et al., 2002; White et al., 2006). Although the parallel effects of *Chmp1a* loss on both MVB structure and ART-EV secretion may be explained by an MVB origin of ART-EVs, additional experiments would be required to definitively determine whether ART-EVs derive from the MVB or the plasma membrane.

### Many Potential EV Functions in the CNS

Whereas the developmental defects caused by the absence of CHMP1A—microcephaly, cerebellar hypoplasia, and short stature—highlight the importance and widespread function of EV-mediated cellular communication during development, the persistence of ART-EVs in the adult human CSF suggests that there may be continued requirements for EV-mediated growth factor secretion in adult brain. The widespread distribution of MVBs along dendrites of pyramidal neurons in the cerebral cortex hints at complex forms of cellular communication, perhaps involving retrograde synaptic signaling. Two recently published papers show that EVs mediate transfer of the synaptic plasticity regulator *Arc* between neurons and from neurons to muscle cells, illustrating the widespread function of EVs in neuronal communication (Pastuzyn et al., 2018; Ashley et al., 2018). In providing a vertebrate model with specific defects in EV function in the CNS, we provide a potential tool to assess the functions of EVs in these diverse and fundamental processes.

## STAR★METHODS

### CONTACT FOR REAGENT AND RESOURCE SHARING

Further information and requests for resources and reagents should be directed to and will be fulfilled by Lead Contact, Christopher A. Walsh (christopher.walsh@childrens.harvard.edu).

### EXPERIMENTAL MODEL AND SUBJECT DETAILS

#### Human Samples

Operating under an approved institutional review board (IRB) protocol, normal human newborn choroid plexus was identified and collected at autopsy by pathologists at Boston Children’s Hospital.

Operating under an approved institutional review board (IRB) protocol, adult human CSF was collected by neurologists at Beth Israel Deaconess Medical Center.

Operating under an approved institutional review board (IRB) protocol, GW20 human fetal whole brain tissue was collected at autopsy by pathologists at Beth Israel Deaconess Medical Center.

#### Animal Use

All animals were cared for humanly and all experiments were approved by Boston Children’s Hospital IACUC. All mice for analysis were collected during embryonic ages or on the day of birth (P0), and because the animals were so young, the sex could not be readily determined.

#### Chmp1a GT mouse generation and mouse breeding

Mouse ES cells with a GT cassette inserted into *Chmp1a* were obtained from BayGenomics (B6;129P2-Chmp1a^Gt(XC472)Byg^/Mmucd) and injected into blastocysts of WT mice. Resulting chimeras were out crossed with WT C57/Bl6 mice to generate heterozygous GT mice. Heterozygous GT mice were backcrossed to C57/Bl6 for 7-8 generations. Mouse DNA was genotyped with the following primers: WT primer F: GAGACAGCGGGTCCGTAAC, WT primer R: AACACACACTCGAACCGAAAG, GT primer F:GAGACA GCGGGTCCGTAAC, GT primer R: GGTCCTAGTCGGAGGTCTCG.

#### Ptch null mouse

*Ptch* null mouse line (Goodrich et al., 1997) was purchased from Jackson Labs and genotyped according to their protocol.

#### CHMP1A null cell line generation

Monoclonal SVG-A *CHMP1A* null line and iPSC *CHMP1A* null lines were generated using CRISPR-Cas9 mutagenesis (Veres et al., 2014) Cas9 was expressed from a plasmid encoding Cas9 and GFP (Gift of Chad Cowan and Kirin Musunuru). gRNA targeting *CHMP1A* was expressed from a co-transfected plasmid, *CHMP1A* protospacer sequence: GAAGGACTCCAAGGCGGAGC. GFP positive cells were grown as single colonies isolated and then sequenced to identify homozygous frameshift mutations in *CHMP1A*. Controls were monoclonal lines from the same experiments shown to be WT by Sanger sequencing. Primers used to genotype were F: GAAGACAGACACTGGAGAAAACC R: CAGAAGACAAACCAGGAGAGTCA.

#### Heterogeneous CHMP1A null cell line generation

SVG-A *CHMP1A* null cell line (heterogenous pool of either WT or KO cells) was generated using CRISPR-Cas9 mutagenesis (Veres et al., 2014). Cas9 was expressed from a lentiviral plasmid (Gift of Feng Zhang, MIT) also encoding the far-red fluorescent protein E2-Crimson, the puromycin resistance gene, and gRNA targeting CHMP1A (GAAGGACTCCAAGGCGGAGC). Lentivirus was harvested 48 h after transfecting the Cas9 packaging plasmids together with plasmids encoding HIV Gag-Pol and VSV-G envelope in Hek293T cells using JetPrime (Polyplus transfection) according to the manufacturer’s instructions. SVG-A cells were incubated with lentivirus for 24 h in the presence of 1 μg/ml Polybrene (EMD Millipore). 3 days later, cells were exposed to puromycin (2 μg/ml) for one week for selection, after which survivors were bulk sorted by FACS based on high E2-Crimson expression. A CRISPR control cell line was generated using a similar strategy and a lentivirus construct encoding gRNA targeting the luciferase gene (CTTCGAAATGTCCGTTCGGT).

#### Cell lines and culture conditions

Human brain astroglial cell line SVG-A and mouse fibroblast line NIH 3T3 were cultured at 37°C and 5% CO2 in high-glucose DMEM (GIBCO) supplemented with 10% FCS and 1% penicillin-streptomycin. iPSCs (line IMR90-4, from WiCell) were grown on Matrigel (BD) in mTESR media (Stem Cell Tech). MEFs were cultured in DMEM high glucose supplemented with 10% FBS, 1% P/S, and 2 mM L-glutamine.

#### Cerebral organoid culture and isolation

Cerebral organoids were harvested at 14 days or 38 days post differentiation, and generated according to the following method: iPSC colonies were grown on a feeder layer of MitC treated mouse embryonic fibroblasts (35,000 cells/cm^2^) and cultured in W0 medium (DMEM/F12, 20% KOSR, 2mM L-glutamine, 1× NEAA, 1× P/S, 50 μM b-mercaptoethanol (Thermofisher) + 8 ng/ml FGF2 (added fresh daily, Sigma). After iPSC colonies reached 2mm in diameter, they were lifted with a cell scraper and transferred to ultra-low attachment 60mm culture dishes (Corning), containing W0 + 1× sodium pyruvate (Differentiation medium, no FGF2). After 24 hours, initial embryoid bodies are formed (defined as day 0) and 50% of medium was replaced with Differentiation medium + small molecule inhibitors to the following final concentrations: 10 μM SB-431542 (Sigma), 1 μM Dorsomorphin (Sigma), 3 μM IWR-1-Endo (Sigma) and 1 μM Cyclopamine (Sigma). Differentiation medium + small molecules was then replaced every other day. On day 4, 60mm plates containing organoids were placed on a hi/lo rocker inside the incubator. On day 18, medium was replaced with Neurobasal/N2 medium (Neurobasal, 1× N2 supplement, 2 mM L-glutamine, 1× P/S) supplemented with 1 μM Cyclopamine. On day 26 medium was replaced with Neurobasal/N2 medium without cyclopamine. At day 35, organoids were cultured in Neurobasal/N2 medium supplemented with 1 μM SAG (treated), or DMSO (control), and refreshed daily. At day 38 organoids were isolated in TRIzol (Thermofisher) for RNA extraction. For immunostainings, organoids were washed twice with PBS and fixed by incubation for 10 minutes in 3.8% PFA / PBS. After fixation, organoids were washed three times in 0.1% BSA / PBS. Then organoids were incubated in 15% sucrose / PBS at 4°C for 2 hours, followed by incubation in 30% sucrose / PBS overnight at 4°C. Using a cut off pipette tip, single organoids were transferred to embedding molds containing Shandon M-1 embedding matrix (Thermofisher) and stored at −80°C.

#### Generation of Chmp1a null MEFs

*Chmp1a* GT heterozygous mice were crossed and E14-15 embryos were collected. Embryos were decapitated and visceral organs removed. The remaining tissue was dissociated with Trypsin and then plated and maintained in DMEM high glucose supplemented with 10% FBS, 1% P/S, and 2 mM L-glutamine. MEFs were assayed before passage 4.

### METHOD DETAILS

#### pH3 analysis

Matching coronal telencephalon sections at E12.5 or E14.5, or midline sagittal cerebellum sections at P0 were immunostained for pH3. pH3+ cells lining the cortical ventricular surface were counted at E14.5, pH3+ cells in the MGE and LGE were counted at E12.5, and pH3+ cells in the cerebellar EGL were counted at P0.

#### LacZ staining

Chemical staining for beta-galactosidase activity was performed with the beta-gal staining kit from Invitrogen (K146501). Briefly, tissue was fixed overnight in2%gluteraldehyde, microtome sectioned (70 uM), and then stained for beta-gal activity according to the kit instructions.

#### *In situ* hybridization

*In situ* hybridization was performed as previously described (Arlotta et al., 2005). *Ptch in situ* probe was a gift from C Cepko and A Joyner. RNA was synthesized with the Megascript kit from Invitrogen. DIG dNTPS and anti-DIG Fab fragments were ordered from Roche.

#### Mouse embryo CSF collection and SHH ELISA

CSF was collected from the 4^th^ ventricle of E14.5-E15.5 *Chmp1a* null mouse embryos and littermate controls using a pulled micropipette. CSF was centrifuged at 10000 G for 5 minutes and then used in the SHH ELISA. SHH ELISA kit was purchased from R and D systems (MSHH00) and used according to the manufacture’s instructions.

#### Human choroid plexus immunostaining

Choroid plexus tissue was fixed in 4% PFA, frozen, and sectioned at 15 um on a cryostat. Cultured iPSCs were fixed with 4% PFA for 10 minutes at RT. Tissue or cells were permeabilized with 0.04% Tween in PBS and blocked in 0.04% tween, 2.5% donkey serum, and 2.5% goat serum in PBS. Sections or cells were incubated with primary antibody diluting in blocking buffer overnight at 4C. Sections or cells were then stained with Alexa secondary antibodies and Hoechst. Imaging was done on a Zeiss 510 confocal microscope.

#### Mouse brain and human organoid immunostaining

Tissue was fixed overnight at 4 C in 4% PFA and sectioned at 70um using a Vibratome. Antigen retrieval was performed with Retrievagen A. Tissue was permeabilized and blocked in 3% BSA, 0.3% Triton X-100,0.3% sodium azide in PBS. Primary antibodies were diluted in blocking buffer and incubated overnight at 4 C. Sections were then stained with Alexa secondary antibodies and Hoechst. Imaging was done on a Zeiss 510 confocal microscope.

Two results confirm the specificity of the CHMP1A antibody used for immunostaining (ProteinTech 15761-1-AP). First, immunoblot of *Chmp1a* null MEF cell lysate with this antibody shows no reactivity (Figure S1B). Second, tissue sections from *Chmp1a* null mice incubated with this antibody show nearly complete loss of signal (Figure S3D).

#### RNAscope

RNAscope on human fetal brain tissue was performed according to manufacturer’s protocol (ACDBio).

#### RNA sequencing library preparation

Cerebral organoid RNA was isolated according to standard TRIzol protocol. RNA was treated with DNaseI (Roche) according to standard protocol for DNA clean-up in RNA samples. RNA was then isolated by column purification (Zymo RNA clean & concentrator 5) and stored at −80°C. For RNA sequencing, first mRNA was isolated from total RNA using polyA selection Dynabeads mRNA DIRECT Micro Purification Kit (Thermofisher). Library was prepared using strand-specific Ion Total RNA-Seq Kit v2 (Thermofisher) and Ion Xpress RNA-Seq Barcode 1-16 (Thermofisher) to label different samples. Sequencing was done using IonProton sequencer, generating single-end reads of around 100bp in length (Thermofisher).

#### Mapping of RNA sequencing data

RNA sequencing data was processed using the Tuxedo package. Briefly, samples were mapped using Tophat2 (Kim et al., 2013), using Bowtie2 (Langmead and Salzberg, 2012) as the underlying alignment tool. The iPSC input fastq files consisted of paired end reads with each end containing 100bp for iPSC data. The cerebral organoid input fastq consisted of single-end reads of around 100bp length for cerebral organoid data. The target genome assembly for these samples was GRCh38/UCSC hg38, and Tophat was additionally supplied with the gene annotation of ENSEMBL84 (GRCh38.p5). Reads mapped per exon were counted using HT-Seq count (union mode) and summed per corresponding gene. HT-Seq count output was normalized using DESeq2, and pairwise comparisons were made to determine significant differences in control and *CHMP1A* null iPSCs or cerebral organoids. Pairwise comparisons were made based on the negative binomial distribution, where the Wald test was used to test for significance. Independent filtering was performed with default settings to correct for multiple testing and the resulting adjusted p values are shown (Love et al., 2014). For generation of heatmaps, z-score was calculated using log-transformed normalized counts. Expression level and fold-change for all mapped transcripts are provided in Table S1.

#### GO pathway enrichment analysis

For genes with at least a 2-fold change between WT and CHMP1a null, enrichment of gene sets for GO- Biological Processes (BP-FAT) and KEGG-pathways was determined by DAVID (Database for Annotation, Visualization and Integrated Discovery) Functional Annotation Clustering (v6.7). For each analysis raw RNaseq read counts were normalized using DEseq2. For week 2 and week 5 respectively, all genes with mean read count > 64 and read count > 32 were included as background. Clusters of GO-terms and KEGG pathways were manually given a name that best represented all individual GO-term categories within each cluster. The DAVID Functional Annotation Tool provides an ‘enrichment score’ but does not provide a measure of statistical significance for Functional Annotation Clusters. For each Functional Annotation Cluster, the benjamini-corrected p value for the highest-ranking individual GO-term in each cluster is displayed next to the bar graphs. GO analysis results and input gene lists are provided in Table S2.

For Figure 4D, the top 250 differentially expressed genes (DESeq2) were selected at d14 or d38 samples from pairwise comparisons between *CHMP1A* null and control cerebral organoids. Upregulated or downregulated genes were placed into separate gene lists. Analysis was done using Panther Overrepresentation test on GO biological processes with Bonferroni correction (Panther release 20170413). Background gene list consisted of all genes, which had on average 10 counts or more mapped in the analyzed samples. In this analysis, the downregulated genes did not show relevant enrichment for brain related GO terms. For Figure S7C, the analysis was also done according to these parameters. Genes that were already differentially expressed between untreated d38 control and d38 *CHMP1A* null organoids were excluded. The remaining genes were selected by DESeq2 adjusted p value < 0.01 instead to limit the amount of genes for analysis.

#### Gene expression correlation human brain and cerebral organoids

Cerebral organoid RNA-seq data was compared to developing human brain RNA-seq data (Brainspan, Allen institute). For Figure 4G, d14 and d38 *CHMP1A* null and control organoids, the top 250 differentially expressed genes (DESeq2) were selected. For Figures S7A and S7B, genes with adjusted p value < 0.01 were selected to limit the number of genes for analysis. Their corresponding expression data was acquired from Brainspan data. Cerebral organoids have best correlation with Brainspan week 9 dorsofrontal cortex data (w9 DFC). Several other time-points of DFC data were used to assess temporal identity of organoids. Genes were ranked by their expression level from high to low, using normalized counts for organoid data, and FPKM for Brainspan data. Then, pairwise calculation of Spearman’s rank correlation was done for each pair and plotted using multi-experiment viewer.

#### Shh signaling in MEFs

MEFs from *Chmp1a* null embryos and littermate controls were cultured as described above. The cells were then cultured in 0.5% FBS with 1 uM SAG for 48 hours. Then total RNA was isolated with RNeasy kit (Ambion) and cDNA was created with Superscript III kit (Invitrogen). Taqman qRT-PCR assay was run on StepOnePlus (Applied Biosystems) with probes for Gli1 and Beta-actin to measure gene expression.

#### Extracellular vesicle isolation

SVG-A cells were either mock-transfected or transfected with a plasmid encoding hSHH using JetPrime according to the manufacturer’s instructions. 24 h later, cells were washed once with PBS and cultured for another 48h in media supplemented with exosome-depleted FBS (System Biosciences) instead of normal FBS, before harvesting cells for lysis and supernatants (SN) for EV isolation. EVs were isolated from conditioned media by differential ultracentrifugation as previously described (Kowal et al., 2016). Briefly, SN was centrifuged for 10 min and 300xg to pellet cells and large debris. The SN was transferred to new tubes and further centrifuged at 2,000xg for 20 min to obtain the 2K pellet. The resulting SN was then transferred to ultracentrifuge tubes and centrifuged in an SW28 rotor (Beckman Coulter) for 40 min at 10,000xg (10K pellet) before a final centrifugation at 100,000xg using the same rotor (100K pellet). Except for the 300xg pellet, all pellets were washed in 5 mL PBS before being recentrifuged at the same speed using a SW55Ti rotor (Beckman Coulter) and finally resuspended in 50 μL cold PBS. All steps were performed at 4°C. The 300xg pellet was pooled with cells harvested from dishes for lysis and WB analysis.

A similar protocol was applied for the isolation of EVs from human CSF (18 mL).

#### Immunoisolation pull-down assays

2 μg of either anti-CD9 (Milipore), anti-CD63 (Pelicluster), anti-Shh (Abcam) or Mouse IgG antibodies were coupled overnight to 1 mg of magnetic Dynabeads using Dynabeads Antibody coupling kit (Invitrogen) according to the manufacturer’s instructions. As described in Kowal et al. (2016), coated beads were washed twice in 1 mL washing buffer (PBS-Tween 0.001%) and incubated overnight using rotation at 4°C with 10 μg of EVs from the 100K pellet resuspended in 500 μL of washing buffer. Beads were washed five times with washing buffer, and, where indicated the unbound material was pooled together with the SN of the washes and centrifuged at 100000xg in a SW55Ti rotor for concentration. Both bound material (PD) and unbound material pellet (flow through [FT]) were resuspended in 30 μL of 2x SDS-PAGE sample buffer (Bio-Rad) and boiled for 5 min at 95°C prior to loading on gel. For MS analysis, Shh pull-down reaction was scaled-up approximately five times.

#### Liquid Chromatography-Tandem Mass Spectrometry (LC-MS/MS)

Samples were lysed using Laemmli buffer (10 mM Tris pH 6,8 ; 1mM EDTA ; 5% β-mercaptoethanol; 5% SDS ; 10% glycerol) and were homogenized for 40 minutes. Samples were briefly centrifuged and the supernatant was collected leaving the magnetic beads in the tubes thanks to a magnet. Samples were then frozen at −80°C until use. Protein concentrations were determined using the RC-DC protein assay (Bio-Rad, Hercules, USA) according to the manufacturer’s instructions using BSA as standard.

50 μL of each sample were concentrated down to 20 μL (SpeedVac, Savant, Thermo Fisher Scientific, Waltham, USA) and heated at 95°C for 5 minutes before being loaded onto an in-house poured 1D SDS-PAGE stacking gel in order to focus proteins into a single “stacked” band. Electrophoresis was performed under a continuous voltage of 50 V for 40 minutes. The proteins were fixed with 50% ethanol and 3% phosphoric acid. After three washes, gels were stained with Colloidal blue.

For each sample, the “stacked” protein-band was excised and cut into four equal pieces. After destaining, DTT reduction and IAM alkylation using an automatic pipetting device (MassPrep, Waters, Milford, MA, USA), proteins were in-gel digested with trypsin (Promega, Madison, WI, USA) overnight at 37°C. Tryptic peptides were extracted first in 60% acetonitrile/0.1% formic acid in water for 90 minutes, followed by a second extraction in 100% acetonitrile for 10 minutes, at 450 rpm on an orbital shaker. Acetonitrile was evaporated under vacuum and samples were adjusted to 8μL using 0.1% formic acid before nanoLC-MS/MS analysis.

NanoLC-MS/MS analyses were performed on a nanoACQUITY Ultra-Performance-LC system (UPLC) coupled to a Q-Exactive Plus Orbitrap (Thermo Fisher Scientific) mass spectrometer.

The Q-Exactive Plus Orbitrap mass spectrometer is equipped with a nanoelectrospray ion source. The UPLC system consisted of a solvent degasser nanoflow pump, a thermostat-controlled column oven set to a temperature of 60°C and a thermostat-controlled autosampler at 10°C. Mobile phase A (99.9% water and 0.1% FA) and mobile phase B (99.9% acetonitrile and 0.1%FA) were delivered at 450 nL/min by the nanoAcquity. Samples were loaded into a Symmetry C18 precolumn (0.18 × 20 mm, 5 μm particle size, Waters) over 3 minutes in 1% buffer B at a flow rate of 5 μL/min. Sample loading was followed by reverse-phase separation at a flow rate of 450 nL/min using an ACQUITY UPLC® BEH130 C18 separation column (200mm × 75 μm id, 1.7 μm particle size, Waters). The Q-Exactive plus Orbitrap instrument was operated in data-dependent acquisition mode by automatically switching between full MS and consecutive MS/MS acquisitions. Survey full scan MS spectra (mass range 300-1,800) were acquired in the Orbitrap at a resolution of 70,000 at 200 m/z with an automatic gain control (AGC) fixed at 3 × 10^6^ ions and a maximal injection time set to 50 ms. The ten most intense peptide ions in each survey scan with a charge state ≥ 2 were selected for MS/MS. MS/MS spectra were acquired at a resolution of 17,500 at 200 m/z, with a fixed first mass at 100 m/z, AGC was set to 1 × 10^5^, and the maximal injection time was set to 100 ms. Peptides were fragmented in the HCD cell by higher-energy collisional dissociation with a normalized collision energy set to 27. Peaks selected for fragmentation were automatically included in a dynamic exclusion list for 60 s, and peptide match selection was turned on. MS data were saved in RAW file format (Thermo Fisher Scientific) using XCalibur. Raw data collected were processed and converted with MSConvert in .mgf peak list format.

Raw files were processed using MaxQuant (v1.5.5.1). Peak lists were searched using the decoy mode of the Andromeda search engine implemented in MaxQuant against a protein database created using MSDA, our home-developed software suite (Carapito et al., 2014). The database contained human and bovine protein sequences (UniProtKB-SwissProt Taxonomy ID: 9606 and 9913 respectively; 26191 entries), which were downloaded in May 2017. Sequences of common contaminants like keratins and trypsin (247 entries, included in MaxQuant) were finally added to the protein database. Regarding search parameters, MS tolerance was set to 20 ppm for the first search and 4.5 ppm for the main search. A maximum number of 2 missed cleavages was accepted, and carbamidomethylation of cysteine residues was set as fixed modification, while acetylation of protein N-termini and oxidation of methionine residues were set as variable modifications. False discovery rates (FDR) were set to 1% for both peptide spectrum matches (minimum length of 7 amino acids) and proteins. Finally, based on the principle of parsimony, shared peptides between proteins were attributed to the protein with the highest number of assigned peptides.

Regarding quantification, data normalization and estimation of protein abundance was performed using the MaxLFQ (label free quantification) option implemented in MaxQuant (Cox et al., 2014). “Match between runs” was enabled using 0.7 minutes time windows after retention time alignment. MaxLFQ quantification was applied using a minimal ratio count of one. Both unmodified and modified (acetylation of protein N-termini and oxidation of methionine residues) peptides were considered for quantification, but shared peptides were not. All other MaxQuant parameters were set as default. The MaxQuant results were visualized and treated with Prostar software for statistical analysis (Wieczorek et al., 2017). Only proteins with at least three intensity values in at least one of the two groups to be compared were retained. Welch’s t test was applied to identify differentially expressed proteins at a p value < 0.05.

#### Western blotting

SVG-A cells were lysed in a buffer containing 50 mM Tris-HCl (pH 7.5), 0.3 M NaCl, 0.5% Triton X-100, and protease inhibitors (1x, Roche) for 20 min on ice, with intense vortexing at the beginning and end of the incubation. Lysates were cleared by centrifugation at 18000xg for 15 min at 4°C before transferring the supernatant to new tubes. The protein content in the lysates and in the purified EV fractions was measured in the presence of 0.2% SDS, using Pierce BCA protein assay kit (Thermo Fisher Scientific) according to the manufacturer’s instructions. For each WB, 20 μg of lysates or 3 μg of EV pellets were loaded on 4%−12% NuPAGE Bis-Tris Protein Gels (Invitrogen) and ran under non-reducing conditions. Transfer was done using iBlot2 NC Transfer stacks (Invitrogen) prior to primary antibody incubation overnight at 4°C. Membranes were revealed by chemiluminescence using Clarity or Clarity Max Western ECL Blotting Substrates (Bio-Rad) and images were acquired using ChemiDoc Touch system (Bio-Rad).

#### Immunostaining and Time-lapse microscopy

SVG-A cells were grown on glass coverslips in 24-well plates for 24 h, prior to transfection with a plasmid encoding for mNeonGreen-Shh using JetPrime according to the manufacturer’s instructions. 24 h later, cells were washed once with PBS and fixed for 20 min at room temperature (RT) with 4% paraformaldehyde. Blocking and permeabilization was done with PBS-0.1% Triton X-100 and 0.5% BSA for 30 min. Cells were incubated with the indicated primary antibodies for 2 h at RT, followed by incubation with secondary antibodies for 45 min, diluted in blocking buffer (Alexa Fluor Donkey anti-Mouse-647 or Alexa Fluor Donkey anti-Rabbit-647). Coverslips were mounted with Fluoromount (Sigma-Aldrich). Image acquisition was performed using an AxioObserver.Z1 inverted microscope (Zeiss) mounted with a spinning disc head (Yokogawa), a back-illuminated EMCCD camera (Evolve, Photometrics) and a X100, 1.45 NA oil objective (Zeiss) controlled by Visiview v.3.3.0 software (Visitron Systems). For live cell imaging, cells were co-transfected as described above with mNG-SHH and TagRFP-CHMP1A on coverslips in 6-well plates, and imaged 48h later in cell media at 37°C and 5% CO_2_ in a dark incubation chamber. Z stacks of 0.5 μm interval with two-channel detection were acquired every 5 s, and time-lapse images were analyzed with Imaris v.9.0.1 (Bitplane).

#### SHH signaling and RT-qPCR

NIH 3T3 cells were seeded in 48-well plates to confluency and cultured for 24 h in complete DMEM. Cells were then grown in media without FBS for another 24h, prior to incubation for another 48 hours with the indicated amounts of SHH-containing 100K vesicles or mock (PBS). As positive control of pathway activation, cells were incubated with 250nM SAG reconstituted in DMSO (Abcam) or DMSO as control. Total RNA was isolated using ReliaPrep RNA Cell Miniprep System (Promega) and cDNA was created with SuperScript VILO Master Mix (Invitrogen) by manufacturer’s instructions. qRT-PCR assay was run on the CFX96 Touch Real-Time PCR Detection System and gene expression was measured with primers specific for the SHH downstream target gene *GLI1* (F: TTGGATTGAACATGGCGTCT and R: CCTTTCTTGAGGTTGGGATGA). Gene expression is shown as fold change relative to mock-treated cells, following normalization to housekeeping gene *RPL27* (F: GTCGAGATGGGCAAGTTCAT and R: GCTTGGCGATCTTCTTCTTG).

#### Cytokinesis analysis

Mouse cortex at E14.5 and cultured iPSCs were fixed in 4% PFA. Cortical ventricular surface or cultured iPSCs were immunostained with antibodies against aurora A kinase, pH3 and Hoechst. Aurora A labels the midbody in anaphase through telophase while pH3 labels histones from prophase through metaphase. A high-power field was imaged and the number of pH3 positive early mitotic cells and the number of aurora A positive late mitotic cells was counted. The ratio of midbodies to pH3 positive cells was calculated as a measure of cytokinesis duration.

#### EGFR degradation assay

WT HeLa cells were transfected with siRNA targeting *CHMP1A* (Thermo, #4392420) or a negative control (Thermo, #4390843) from Ambion Silencer Select according to the manufacturer’s protocol. Cells were grown for 48 hours, and then placed in serum free media for 2 hours. EGF was added to the media at 250 ng/mL and cells were fixed at 0 and 2 hours. Cells were permeabilized in PBS, 0.5% BSA, 0.05% saponin, and stained with anti EGFR antibody (13A9, Genentech) for 1 hour and then Alexa fluorescent secondary for 30 minutes. Fluorescence was then quantified at the single cell level for hundreds to thousands of cells using a BD FACS Aria flow cytometer.

#### Electron microscopy

Embryonic mouse choroid plexus and P0 mouse cerebellum was fixed in 2.5% Glutaraldehyde and 2% Paraformaldehyde in 0.1 M sodium cacodylate buffer (pH 7.4) at 4C overnight. 70 um thick tissue sections were cut on a vibratome. Sections were washed in 0.1 M cacodylate buffer and postfixed with 1% Osmiumtetroxide (OsO4) and 1.5% Potassiumferrocyanide (KFeCN6) for 1 hour, washed in water 3x and incubated in 1% aqueous uranyl acetate for 1 hour followed by 2 washes in water and subsequent dehydration in grades of alcohol (10 min each; 50%, 70%, 90%, 2× 10 min 100%). The samples were then infiltrated for 15 min in a 1:1 mixture of propyleneoxide and TAAB Epon (Marivac Canada Inc. St. Laurent, Canada). The samples were embedded in drops of TAAB Epon between two sheets of aclar plastic (Electron Microscopy Sciences) and polymerized at 60C for 48 hours. Ultrathin sections (about 80 nm) were cut on a Reichert Ultracut-S microtome, placed onto copper grids, stained with uranyl acetate and lead citrate and examined in a JEOL 1200EX transmission electron microscope. Images were recorded with an AMT 2k CCD camera.

SEM samples were postfixed in 1.0% osmium tetroxide in 0.1M cacodylate buffer (pH 7.4) for 1 hour at room temperature. Following postfixation, the samples were rinsed with buffer then dehydrated through a graded series of ethanol. The specimens were then critical point dried with CO2 using a Samdri PVT-3 critical point dryer (Tousimis Corp. Rockville, MD). The specimens were attached to specimen mounts using conductive adhesive tabs, coated with 5nm platinum using a Cressington 208HR sputter coater (Cressington Scientific Instruments, Ltd. Walford, UK). SEM images were collected on a Hitachi S-4800 at Northeastern University.

For FIB-SEM, Durcupan embedded sample was first mounted on a Cu stud, then imaged by a customized Zeiss NVision40 FIB-SEM system previously described (Xu et al., 2017). The sample was biased at 400 V to improve image contrast by filtering out secondary electrons. The block face was imaged by a 1 nA electron beam with 1.5 keV landing energy at 500 kHz. The x-y pixel resolution was set at 8 nm. A subsequently applied focused Ga^+^ beam of 27 nA at 30 keV strafed across the top surface and ablated away 2 nm of the surface. The newly exposed surface was then imaged again. The ablation – imaging cycle continued about once every minute for multiple days. The sequence of acquired images formed a raw imaged volume, followed by post processing of image registration and alignment using a Scale Invariant Feature Transform (SIFT) based algorithm. The aligned stack was binned by a factor of 4 along z to form a final isotropic volume of 28×34×5 μm^3^ with 8×8×8 nm^3^ voxels, which can be viewed in arbitrary orientations.

### QUANTIFICATION AND STATISTICAL ANALYSIS

In all analyses, mean values are presented for pooled data and errors bars are SEM.

Relative intensities of the WB bands were quantified using ImageLab software version 5.2.1 (Bio-Rad) using Volume Tools, global background subtraction and linear regression method. Band intensities of WT samples were set as 100% and used to calculate the protein levels in the KO samples, plotted as percentage of WT (Figures 6E and 6F).

For mass-spec experiments, Welch’s t test was applied to identify differentially expressed proteins at a p value < 0.05 (Figure 7E, Table S3).

For RNA-sequencing of cerebral organoids, the significance of gene expression differences was calculated with the DESeq2 adjusted p value. For GO pathway analysis, each Functional Annotation Cluster, the benjamini-corrected p value for the highest-ranking individual GO-term in each cluster is displayed next to the bar graphs. For comparison between organoid gene expression and human developing cortex, pairwise calculation of Spearman’s rank correlation was done for each comparison.

For all other quantifications, statistical significance was determined using a two-tailed, unpaired t test or a Mann-Whitney test. All counts of ILVs per MVB failed the D’Agostino and Pearson normality test (p < 0.01). As a result, the nonparametric Mann-Whitney test was used to test significance of ILV per MVB differences. Statistical analyses were performed using GraphPad Prism version 7.

### DATA AND SOFTWARE AVAILABILITY

RNA sequencing data have been deposited in GEO (GSE115867).

Proteomics data have been deposited in ProteomeXchange (PXD007990).

## Supplementary Material

1

2

3

4

5

## Figures and Tables

**Table:** KEY RESOURCES TABLE

REAGENT or RESOURCE Antibodies	SOURCE	IDENTIFIER
Rat anti L1CAM	Millipore	MAB5272
Mouse anti TUJ1	Biolegend	801201
Rabbit anti CHMP1A (WB)	Stan Hollenberg	N/A
Rabbit anti CHMP1A (IHC)	ProteinTech	15761-1-AP
Mouse anti Beta actin	Abcam	AC-15
Mouse anti Calbindin	Abcam	AF2E5
Mouse anti AQP1	Santa Cruz	SC25287
Rat anti CTIP2	Abcam	AB18465
Goat anti SOX2	Santa Cruz	SC17330
Mouse anti CD63	Pelicluster	M1544
Rabbit anti CUX1	Santa Cruz	SC13024 M-222
Rat anti phospho Histone H3	Sigma	H9908
Rabbit anti SHH	Abcam	AB73958
Rabbit anti Cleaved caspase 3	Abcam	AB13847
Mouse anti Beta catenin	BD	610153
Rabbit anti Atypical PKC	Santa Cruz	SC216
Mouse anti Aurora A	BD	611082
Mouse anti CD9	Millipore	CBL162
Rabbit anti CD9	Abcam	ab92726
Mouse anti CD81	Santa Cruz	sc-166029
Mouse anti CD81	Abcam	ab59477
Mouse anti TSG101	Abcam	ab83
Mouse anti TSG101	Thermo Fisher	MA1-23296
Mouse anti EGFR	Genentech	13-A9
Rabbit anti Syntenin	Abcam	ab133267
Rat anti GP96	Enzo Life Sciences	ADI-SPA-850-F
Rabbit anti AXL	Cell Signaling	8661S
Rabbit anti RAB18	MilliporeSigma	SAB4200173
Rabit anti TMED10	Sigma-Aldrich	HPA047139
Goat anti SHH	Santa Cruz	sc-1194
Rabbit anti PAX6	Covance	PRB-278P-100

Biological Samples		

Human CSF	Eric Wong	N/A
Human choroid plexus	Hart Lidov	N/A

Chemicals, Peptides, and Recombinant Proteins		

Smoothened agonist (SAG)	Abcam	ab142160

Critical Commercial Assays		

Dynabeads Antibody Coupling Kit	Invitrogen	14311D
RNAscope	ACDBio	N/A
Shh ELISA	R&D Systems	MSHH00

Deposited Data		

RNA sequencing	GEO	GSE115867
Proteomics	ProteomeXchange	PXD007990
Experimental Models: Cell Lines		
SVG-A	T Kirchhausen	N/A
iPSC (IMR90)	WiCell	N/A
*NIH 3T3*	ATCC	N/A
Chmp1a null MEF	This paper	N/A

Experimental Models: Organisms/Strains		

Chmp1a GT mouse	This paper	N/A
Ptch mutant mouse	JAX	N/A

Oligonucleotides		

CHMP1A gRNA	This paper	N/A
Luciferase gRNA	This paper	N/A
Gli1 qPCR F primer	This paper	N/A
Gli1 qPCR R primer	This paper	N/A
Rpl27 qPCR F primer	This paper	N/A
Rpl27 qPCR R primer	This paper	N/A
Chmp1a WT F primer	This paper	N/A
Chmp1a GT F primer	This paper	N/A
Chmp1a WT R primer	This paper	N/A
Chmp1a GT R primer	This paper	N/A

Recombinant DNA		

Mouse Ptch *in situ* probe	Constance Cepko	N/A
mNG-SHH	Corey Harwell	N/A
TagRFP-CHMP1A	This paper	N/A
Cas9 GFP	Kirin Musunuru	N/A
Cas9-E2-Crimson	Feng Zhang	N/A
gRNA backbone	Kirin Musunuru	N/A
*CHMP1A* siRNA	Thermo	4392420
Control siRNA	Thermo	4390843
RNAscope probe Hs CD63	ACDBio	505901
RNAscope probe Hs SHH	ACDBio	600951
RNAscope probe Hs PTCH	ACDBio	422161
RNAscope probe Hs CHMP1A	ACDBio	505911

Software and Algorithms		

Visiview	Visitron Systems	N/A
Imaris	Bitplane	N/A
ImageLab	Bio-Rad	N/A
CFX Manager	Bio-Rad	N/A
ImageJ	NIH	N/A
XCalibur	Thermo	N/A
MSConver	N/A	N/A
MaxQuant	N/A	N/A
Prostar	N/A	N/A
Prism 7	GraphPad	N/A

## Highlights

- *CHMP1A* is required for progenitor proliferation in developing brain
- Loss of *CHMP1A* decreases sonic hedgehog secretion
- Loss of *CHMP1A* decreases intraluminal vesicles within multivesicular bodies
- *CHMP1A* regulates SHH secretion on the extracellular vesicle subtype ART-EV
