# Supplementary material for: The ESCRT-III Protein CHMP1A Mediates Secretion of Sonic Hedgehog on a Distinctive Subtype of Extracellular Vesicles
Source: Cell Rep. Author manuscript; Available in PMC 2018 Oct 10. (PMC6178983; doi:10.1016/j.celrep.2018.06.100)
Supplement: 1 [file NIHMS1503267-supplement-1.pdf]

Cell Reports, Volume *24*

Supplemental Information

The ESCRT-III Protein CHMP1A Mediates Secretion of Sonic Hedgehog on a Distinctive Subtype of Extracellular Vesicles

Michael E. Coulter, Cristina M. Dorobantu, Gerrald A. Lodewijk, François Delalande, Sarah Cianferani, Vijay S. Ganesh, Richard S. Smith, Elaine T. Lim, C. Shan Xu, Song Pang, Eric T. Wong, Hart G.W. Lidov, Monica L. Calicchio, Edward Yang, Dilenny M. Gonzalez, Thorsten M. Schlaeger, Ganeshwaran H. Mochida, Harald Hess, Wei-Chung Allen Lee, Maria K. Lehtinen, Tomas Kirchhausen, David Haussler, Frank M.J. Jacobs, Raphael Gaudin, and Christopher A. Walsh

Supplemental Figures and Text for:

The ESCRT-III protein CHMP1A mediates secretion of sonic hedgehog on a distinctive subtype of extracellular vesicles

Michael E. Coulter[1,2‡], Cristina M. Dorobantu[3‡], Gerrald A. Lodewijk[4‡], François Delalande[5], Sarah Cianferani[5], Vijay Ganesh[1,6,7], Richard S. Smith[1], Elaine T. Lim[1], C. Shan Xu[8], Song Pang[8], Eric T. Wong[9], Hart G.W. Lidov[10], Monica L. Calicchio[10], Edward Yang[11], Dilenny M. Gonzalez[1], Thorsten M. Schlaeger[12], Ganeshwaran H. Mochida[1,7], Harald Hess[8], Wei-Chung Allen Lee[13], Maria K. Lehtinen[10], Tomas Kirchhausen[14,15], David Haussler[16], Frank M.J. Jacobs[4*], Raphael Gaudin[3,14*], Christopher A. Walsh[1*]

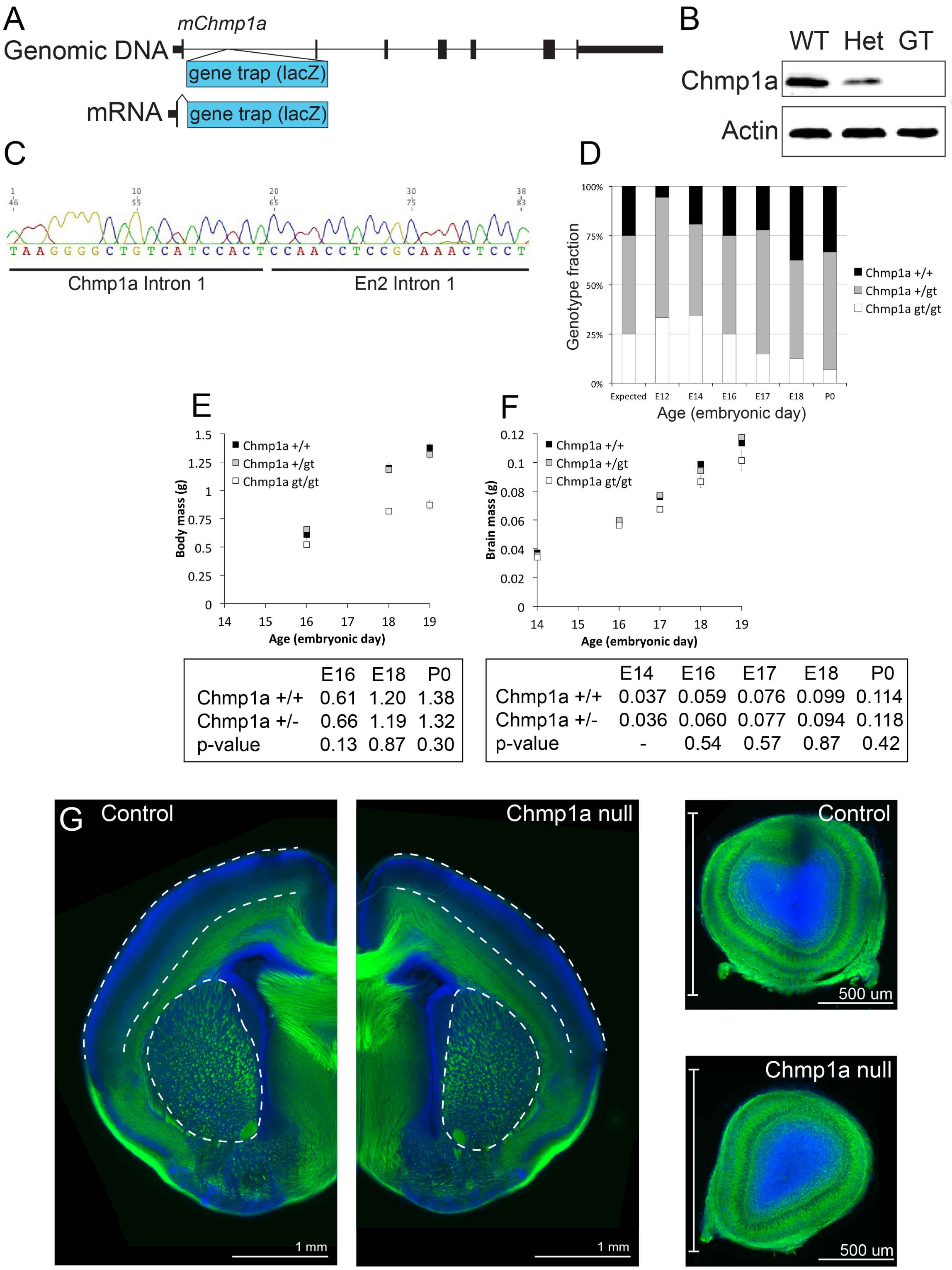

	E16	E18	P0
Chmp1a +/+	0.61	1.20	1.38
Chmp1a +/-	0.66	1.19	1.32
p-value	0.13	0.87	0.30

	E14	E16	E17	E18	P0
Chmp1a +/+	0.037	0.059	0.076	0.099	0.114
Chmp1a +/-	0.036	0.060	0.077	0.094	0.118
p-value	-	0.54	0.57	0.87	0.42

Figure S1. *Chmp1a* gene trap mouse model, Related to Figure 1

(A) Model of gene trap cassette in mouse *Chmp1a* locus. (B) Western blot shows *Chmp1a* null mice express no Chmp1a protein. (C) Sanger sequencing of genomic DNA in *Chmp1a* null mice shows fusion of *Chmp1a* intron 1 to *En2* intron 1 in GT cassette. (D) Mendelian ratios of *Chmp1a* GT litters during embryonic development. Expected Mendelian ratio is shown in the left bar. At late embryogenesis, *Chmp1a* gt/gt begin to die and few are recovered at P0. (E) *Chmp1a* +/gt embryos do not have a defect in embryonic development compared to *Chmp1a* +/+ embryos. Mass and p-values of +/+ vs +/gt are shown below, two-tailed t-test. (F) *Chmp1a* +/gt embryos do not have a defect in embryonic brain development compared to *Chmp1a* +/+ embryos. Mass and p-values of +/+ vs +/gt are shown below, two-tailed t-test. (G) *Chmp1a* null mouse telencephalon and olfactory bulb are hypomorphic.

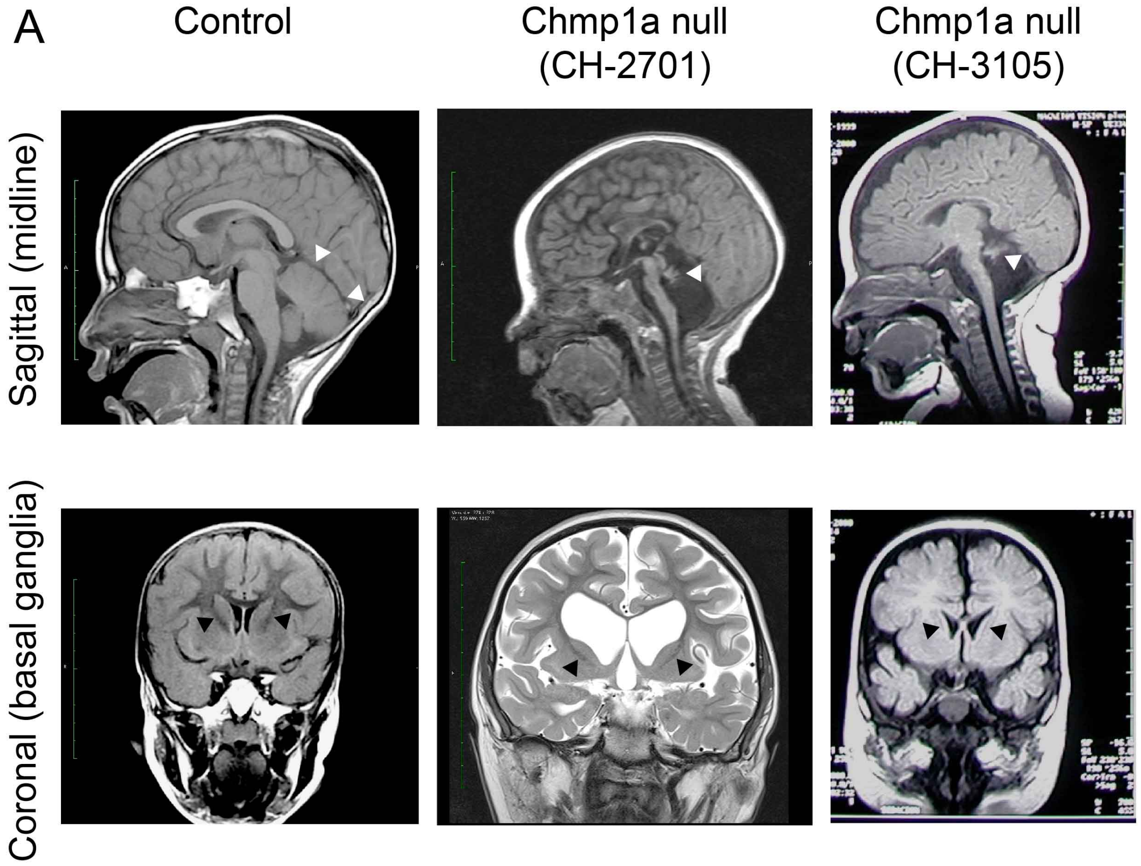

Figure S2. MRI imaging of patients with *CHMP1A* null mutations, Related to Figure 1

(A) MRI images of one control and two patients with *CHMP1A* null mutations. Control is a neurologically normal 2 year old male, CH-2701 is 6 month old female with p.Gln30* *CHMP1A* mutation, and CH-3105 is 3 month old male with c.28-13G>A *CHMP1A* mutation (Mochida et al., 2012). Sagittal images show decreased head circumference (microcephaly) and severe cerebellar hypoplasia (arrowhead) in absence of *CHMP1A*. Coronal images show hypoplasia of caudate (arrowheads) in absence of *CHMP1A*.

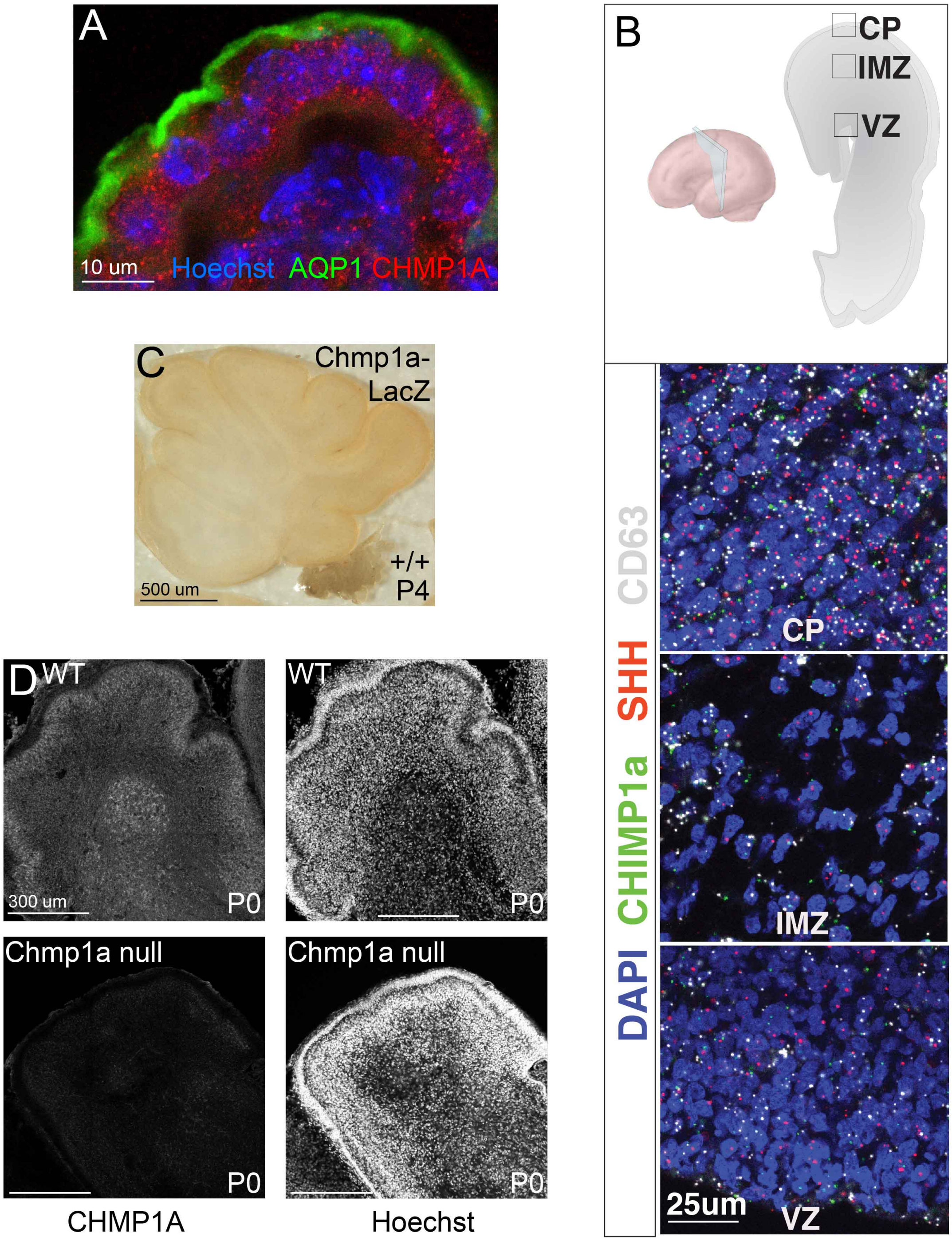

Figure S3. ***CHMP1A*** **RNAscope, ChP expression, and expression controls, Related to Figure 2**

(A) Chmp1a immunoreactivity is punctate in epithelial cells of hindbrain choroid plexus at P0. AQP1 labels ventricular surface. (B) *CHMP1A*, *SHH*, and *CD63* expression in developing human cortex by RNAscope. (C) Beta-gal staining in wild type mouse shows no signal in

cerebellum or choroid plexus at P4. (D) Immunostain for Chmp1a shows no signal in *Chmp1a* null cerebellum at P0. All panels are representative image of ≥2 experiments.

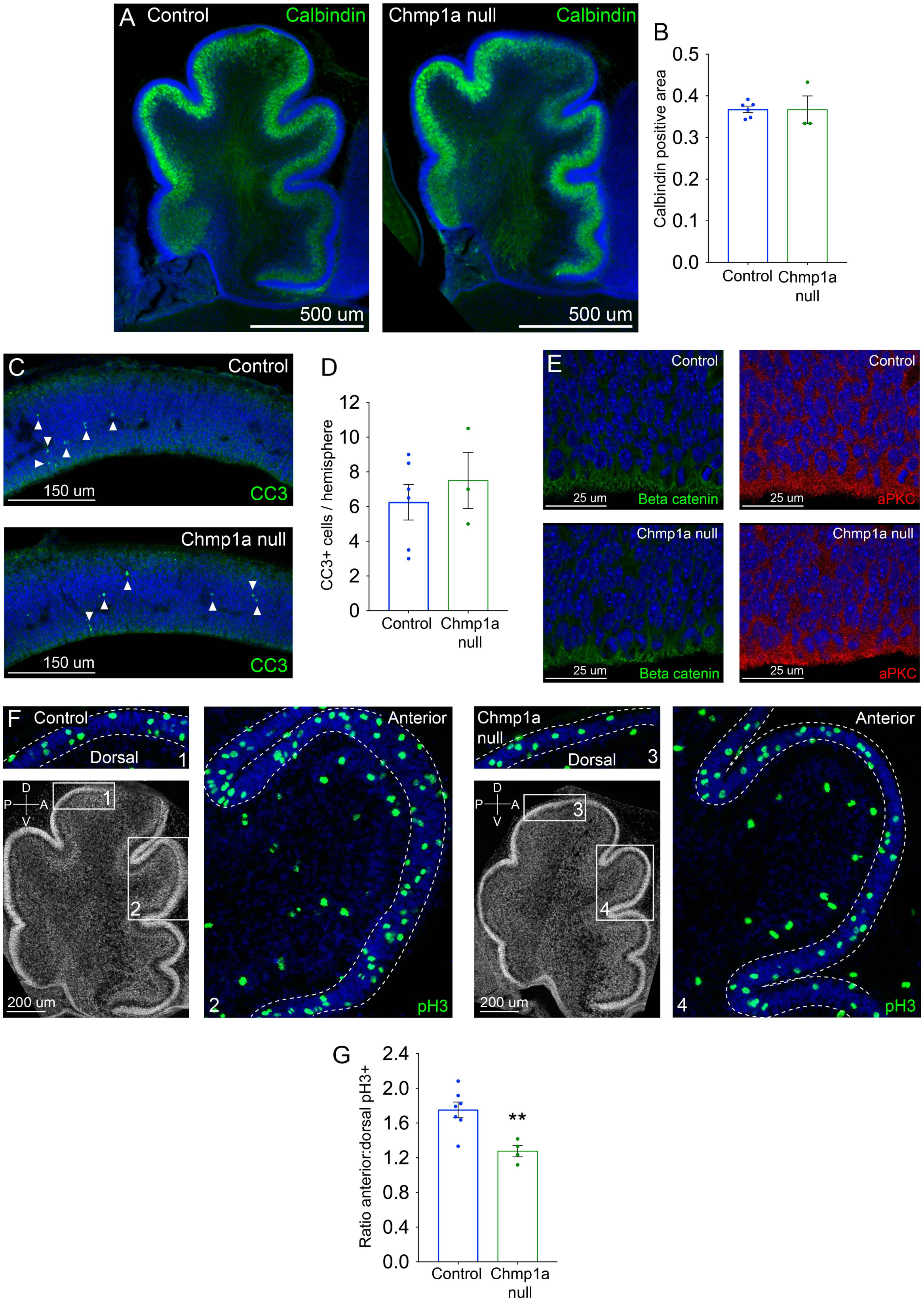

A Control
Calbindin
Chmp1a null
Calbindin
500 um
500 um
B
Calbindin positive area
0.5
0.4
0.3
0.2
0.1
0.0
Control
Chmp1a null
C
Control
150 um
CC3
Chmp1a null
150 um
CC3
D
CC3+ cells / hemisphere
12
10
8
6
4
2
0
Control
Chmp1a null
E
Control
25 um
Beta catenin
Control
25 um
aPKC
Chmp1a null
25 um
Beta catenin
Chmp1a null
25 um
aPKC
F
Control
Dorsal
1
D
P
A
V
1
2
200 um
Anterior
2
pH3
Chmp1a null
Dorsal
3
D
P
A
V
3
4
200 um
Anterior
4
pH3
G
Ratio anterior:dorsal pH3+
2.4
2.0
1.6
1.2
0.8
0.4
0.0
**
Control
Chmp1a null

Figure S4. Developing cortex and cerebellum in *Chmp1a* null mouse, Related to Figure 3

(A) Purkinje cell layer is intact in *Chmp1*a null P0 pups and unchanged compared to littermate controls. Immunostain for Calbindin labels Purkinje cells in control and *Chmp1a* null P0 cerebellum. (B) Fraction of cerebellum area covered by the Purkinje cell layer has no detectable difference in *Chmp1a* null pups compared to controls (Control: n=6, *Chmp1a* null: n=3, two-tailed t-test, p = 0.97). (C) During cortical development, there is no detectable increase in apoptosis in *Chmp1a* null embryos compared to controls. Apoptotic cells in developing cortex (E12.5) were labeled with immunostaining for cleaved caspase 3. (D) Quantification of (A), Control: n=6, *Chmp1a* null: n=3, two-tailed t-test, p = 0.52. (E) There is no detectable defect in cortical ventricular surface formation in *Chmp1a* null embryos. Immunostaining for beta-catenin and atypical protein kinase C, two proteins that localizes at the ventricular surface, showed no focal defects in ventricular surface or changes in protein distribution in *Chmp1a* null developing cortex (E14.5). Representative image of 2 experiments. (F) SHH drives GCP proliferation more in anterior lobe than in dorsal lobe. (G) Control littermates have 75% more mitotic GCPs in the anterior lobe compared to the dorsal lobe; in contrast, *Chmp1a* null pups have only 28% more mitotic GCPs in anterior lobe, Control: n=7, *Chmp1a* null: n=4, two-tailed t-test, p = 0.005. Two-tailed, unpaired t-test, ** p < 0.01.

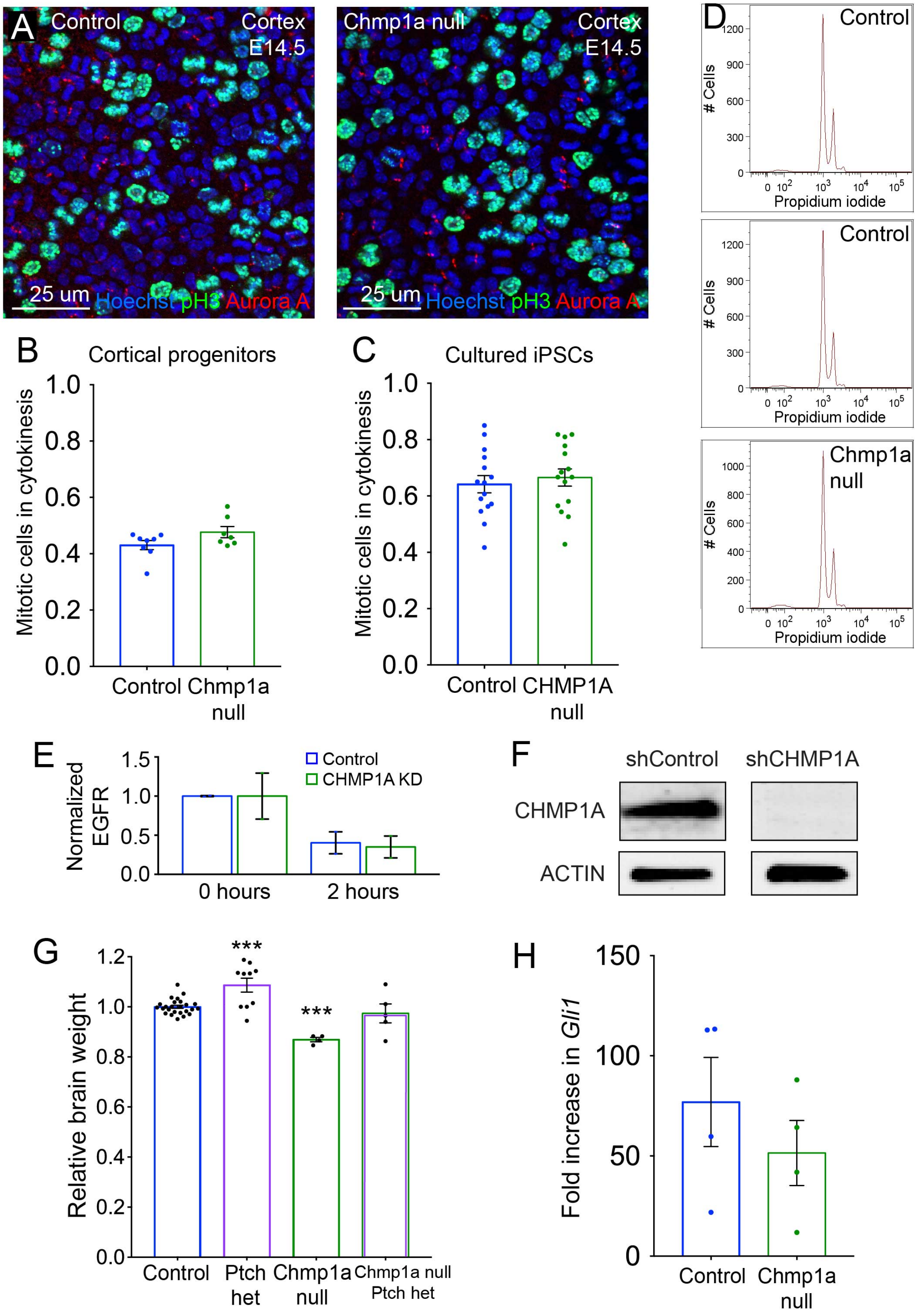

A
Control
Cortex E14.5
25 um
Hoechst pH3 Aurora A
Chmp1a null
Cortex E14.5
25 um
Hoechst pH3 Aurora A
B
Cortical progenitors
Mitotic cells in cytokinesis
Control
Chmp1a null
C
Cultured iPSCs
Mitotic cells in cytokinesis
Control
CHMP1A null
D
Control
Cells
Propidium iodide
Control
Cells
Propidium iodide
Chmp1a null
Cells
Propidium iodide
E
Normalized EGFR
Control
CHMP1A KD
0 hours
2 hours
F
shControl
shCHMP1A
CHMP1A
ACTIN
G
Relative brain weight
***
***
Control
Ptch het
Chmp1a null
Chmp1a null Ptch het
H
Fold increase in Gli1
Control
Chmp1a null

Figure S5. ***Chmp1a* is not required for other ESCRT functions and *Chmp1a* null ; *Ptch* mutant mouse and *Gli1* in MEFs, Related to Figure 3**

(A) Cytokinesis disruption was not detected in the absence of CHMP1A. Ventricular surface of mouse cortex and cultured iPSCs were immunostained for pH3 and aurora A (images show mouse cortex ventricular surface). pH3 labels early mitotic cells and aurora A labels the midbody of late mitotic cells. The fraction of mitotic cells in cytokinesis was calculated as: (Aurora A) / (Aurora A + pH3). (B) There was no detectable difference in the fraction of cells in cytokinesis in ventricular cortical progenitors of *Chmp1a* null embryos compared to littermate controls (Control: n=8, *Chmp1a* null: n=7, two-tailed t-test, p = 0.093). (C) There was no detectable difference in fraction of cells in cytokinesis in *CHMP1A* null and WT cultured iPSCs (Control: n=15, *Chmp1a* null: n=15, two-tailed t-test, p = 0.59). (D) Propidium iodide staining of *Chmp1a* null and control MEF lines. There was no detectable difference in distribution of DNA content between *Chmp1a* null (n=1) and control MEFs (n=2). (E) There was no detectable difference in EGFR degradation in *CHMP1A* depleted HeLa cells compared to control. 0 hours: Control, n=2, *CHMP1A* KD, n=2; 2 hours: Control n=2, *CHMP1A* KD, n=2. EGFR remaining at 2 hours, Control: 40%, *CHMP1A* KD: 35%, two-tailed t-test, p = 0.82. (F) *CHMP1A* siRNA depletes all CHMP1A detected by immunoblot. (G) Brain weight of *Chmp1a* null ; *Ptch* heterozygous mouse embryos at E18.5/P0 (Control: n=25, *Ptch* het: n=10, *Chmp1a* null: n=4, Chmp1a null ; *Ptch* het: n=5, two-tailed t-test, *Ptch* het: p < 0.001, *Chmp1a* null: p < 0.001, *Chmp1a* null ; *Ptch* het: p = 0.22). (H) *Shh* signaling is intact in *Chmp1a* null MEFs. MEFs were stimulated with SAG and *Gli1* expression increase was used to measure *Shh* pathway activation. There is no detectable difference between *Chmp1a* null and littermate control MEFs (Control: n=4, *Chmp1a* null: n=4, two-tailed t-test, p = 0.39). Two-tailed, unpaired t-test, *** p < 0.001.

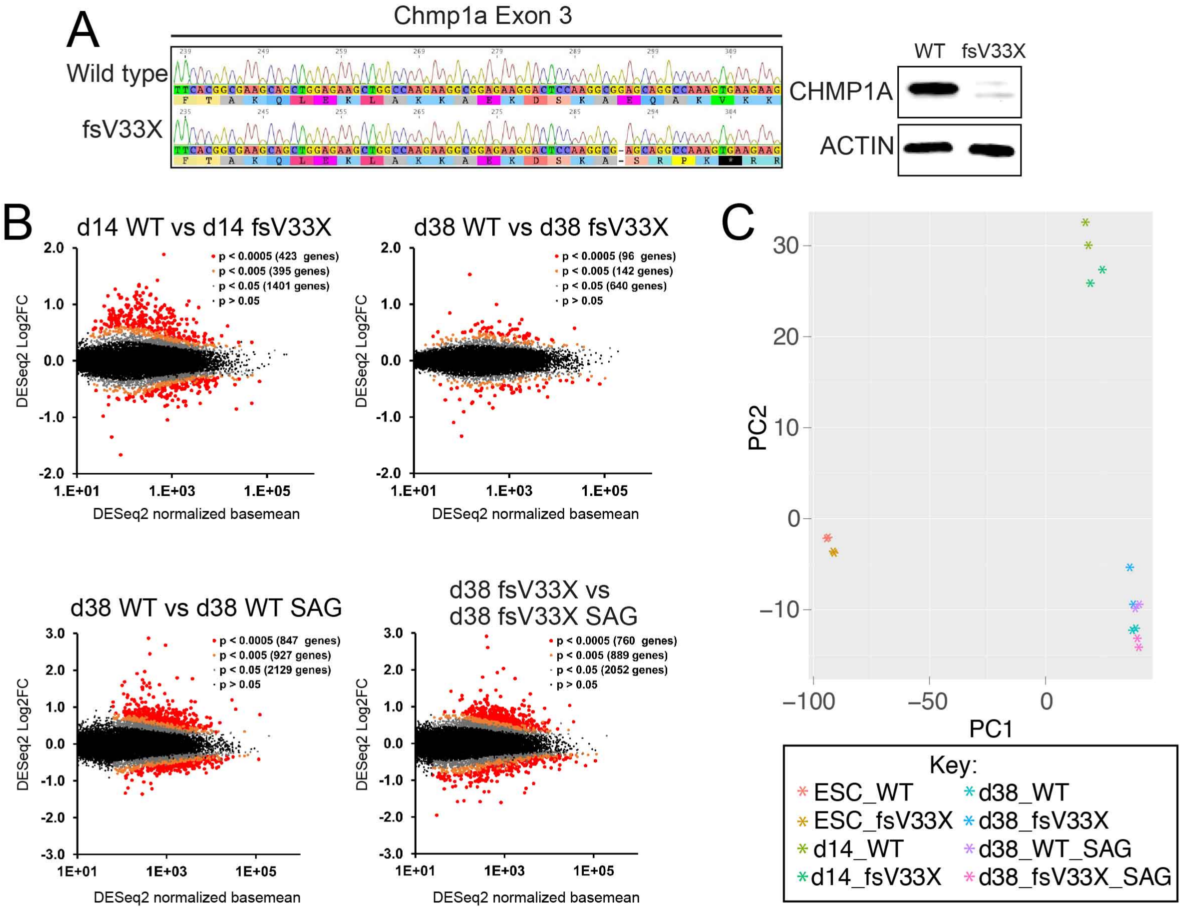

Figure S6. Human organoid RNA sequencing quality control metrics, Related to Figure 4

(A) Sanger sequencing showing wild type and 1 bp frameshift deletion and resulting premature STOP codon in *CHMP1A* null iPSC line (fsV33X). *CHMP1A* null iPSCs do not express CHMP1A protein. (B) MA plots of pairwise comparisons of organoid RNA-sequencing data. Data shows output values of DESeq2 analysis, Y-axis shows DESeq2 corrected Log2 fold-change, X-axis basemean expression levels. Colored dots represent genes according to their p-value. (C) PCA plot of all samples and replicates used in this study after normalization using DESeq2.

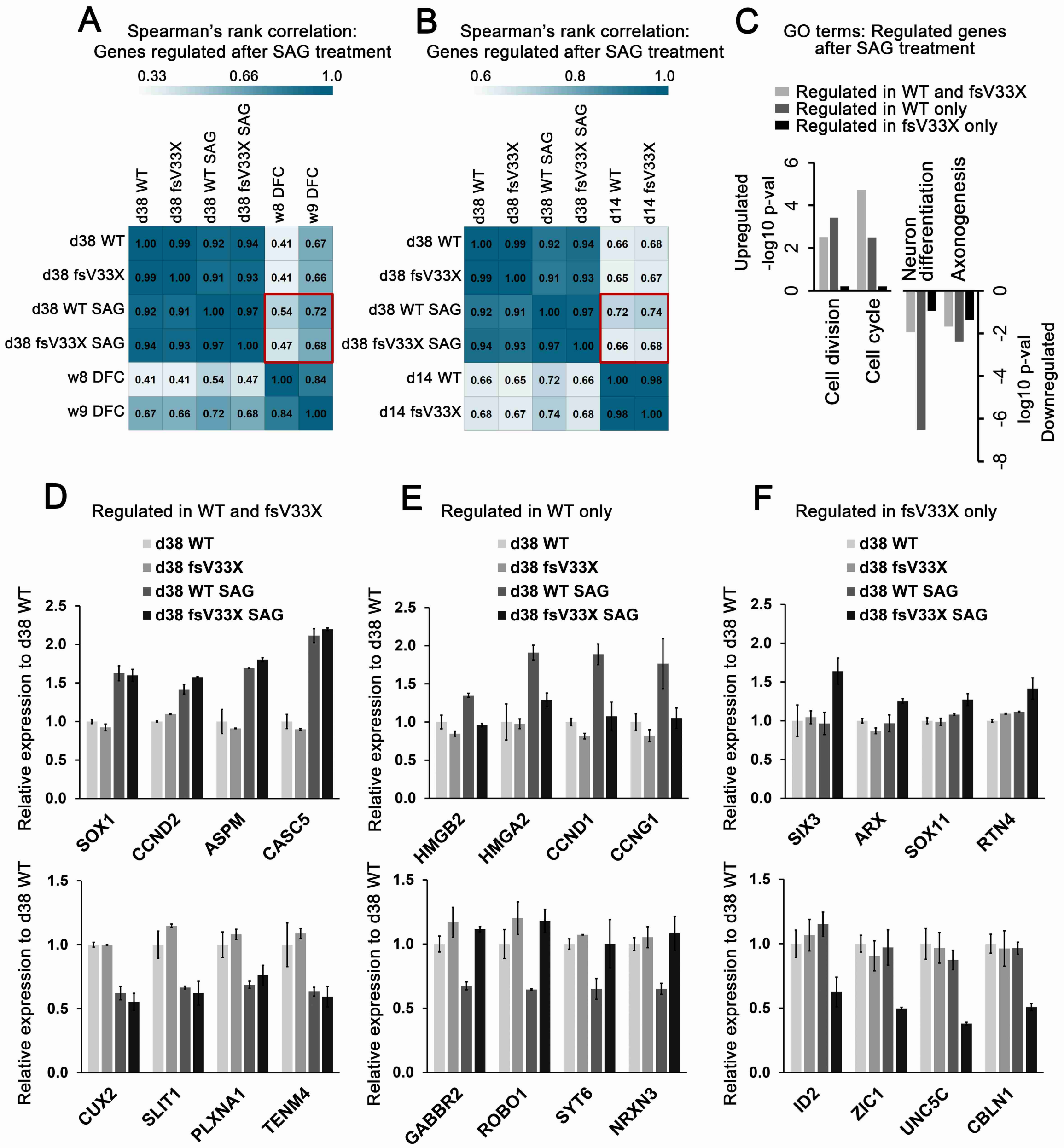
A
Spearman's rank correlation: Genes regulated after SAG treatment
B
Spearman's rank correlation: Genes regulated after SAG treatment
C
GO terms: Regulated genes after SAG treatment
Regulated in WT and fsV33X
Regulated in WT only
Regulated in fsV33X only
Upregulated -log10 p-val
Downregulated log10 p-val
Cell division
Cell cycle
Neuron differentiation
Axonogenesis
D
Regulated in WT and fsV33X
E
Regulated in WT only
F
Regulated in fsV33X only
d38 WT
d38 fsV33X
d38 WT SAG
d38 fsV33X SAG
w8 DFC
w9 DFC
d14 WT
d14 fsV33X
Relative expression to d38 WT
SOX1
CCND2
ASPM
CASC5
HMGB2
HMGA2
CCND1
CCNG1
SIX3
ARX
SOX11
RTN4
CUX2
SLIT1
PLXNA1
TENM4
GABBR2
ROBO1
SYT6
NRXN3
ID2
ZIC1
UNC5C
CBLN1

Figure S7. Gene expression changes following SAG treatment of human organoids, Related to Figure 4

(A) Comparison of d38 organoid data to Brainspan data using Spearman's rank correlation. Highlighted in red shows SAG treatment of control organoids increases correlation with w9 and more strongly with w8 DFC data compared to DMSO treated control organoids. *CHMP1A* null organoids respond as well but to a lesser extent. (B) Comparison of d38 organoid data to d14 organoid data. Similarly to panel A, SAG treated control organoids show better correlation with early stage day14 organoids. (C) GO analysis of differentially expressed genes. SAG treatment leads to upregulation of proliferation genes and downregulation of differentiation genes in both control and *CHMP1A null* cerebral organoids (light grey bars). There are also genes regulated only in control or only in *CHMP1A* null organoids, which were analyzed as separate groups (dark grey bars, black bars respectively). Control organoids show extra sets of genes positively regulating cell cycle, and negatively regulating differentiation. (D), (E), (F) Representative genes and their expression changes from analyses and groups shown in panels A, B and C.

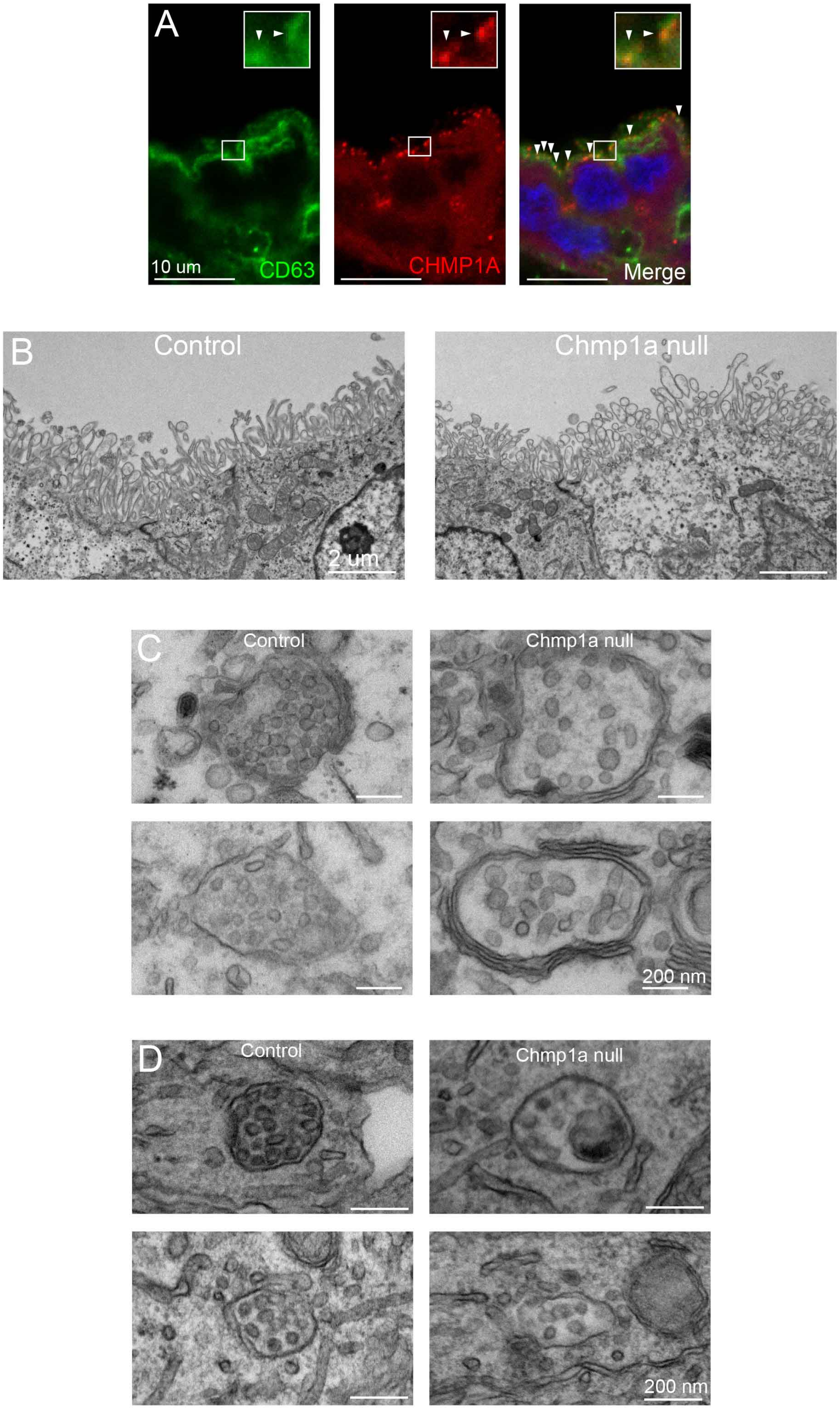
A
10 um
CD63
CHMP1A
Merge
B
Control
Chmp1a null
2 um
C
Control
Chmp1a null
200 nm
D
Control
Chmp1a null
200 nm

Figure S8. Choroid plexus epithelial surface and additional MVBs, Related to Figure 5

(A) Partial colocalization of CHMP1A and CD63 puncta (inset and arrowheads) at the ventricular surface of human hindbrain ChP (11 d.o.). Representative image of 2 experiments. (B) There is no detectable defect in choroid plexus epithelial cell microvilli in *Chmp1a* null embryos at E17.5. (C) Additional examples of choroid plexus epithelial cell MVBs in control and *Chmp1a* null littermates. *Chmp1a* null MVBs contain fewer ILVs and often contain large ILVs. (D) Additional examples of Purkinje cell MVBs from *Chmp1a* null P0 pups and littermate controls. *Chmp1a* null MVBs contain fewer ILVs than controls.

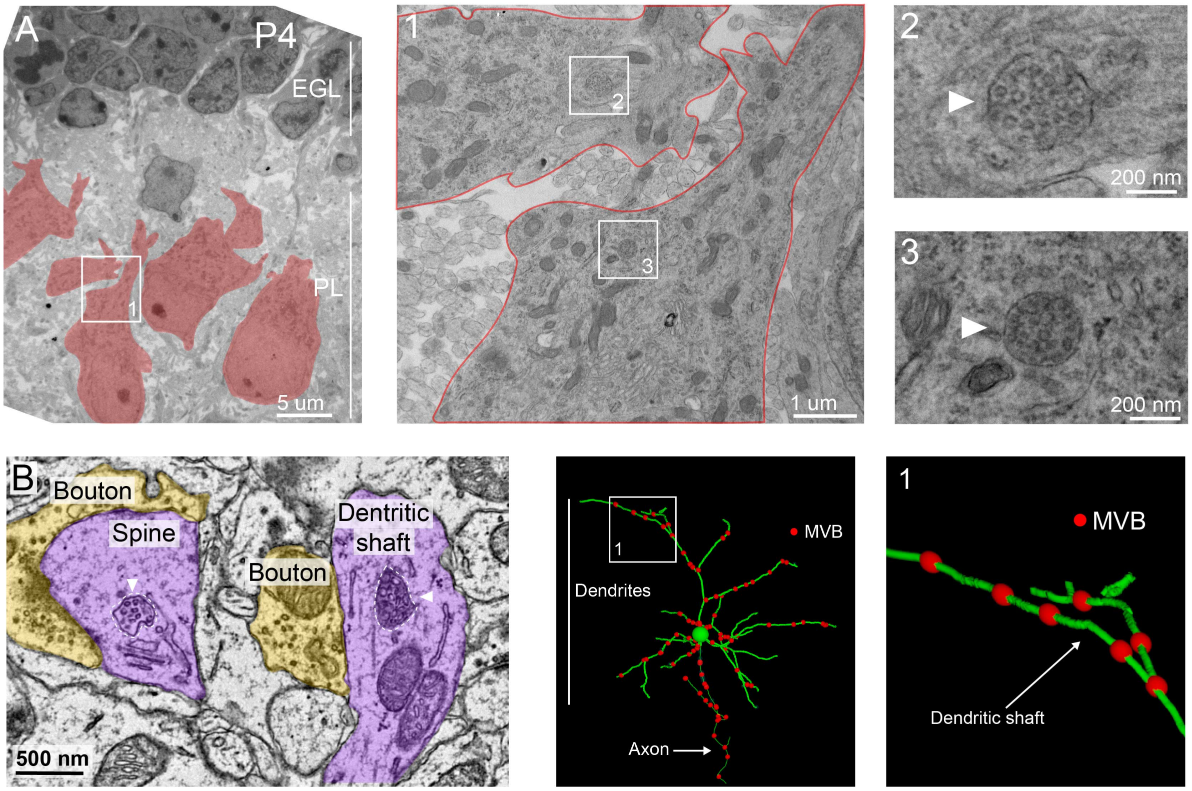

Figure S9. MVBs localize to dendrites in postmitotic neurons, Related to Figure 5

(A) MVBs are in distal (2) and proximal (3) Purkinje cell dendrites in developing cerebellum at P4. (B) MVBs in dendrites of layer II/III cortical pyramidal neurons. High magnification image shows a MVB (white arrowhead) near an excitatory synapse in a pyramidal cell dendritic spine and near an inhibitory synapse in a dendritic shaft. MVBs locations are represented by red dots in a rendered 3D TEM reconstruction of a serially sectioned pyramidal neuron.

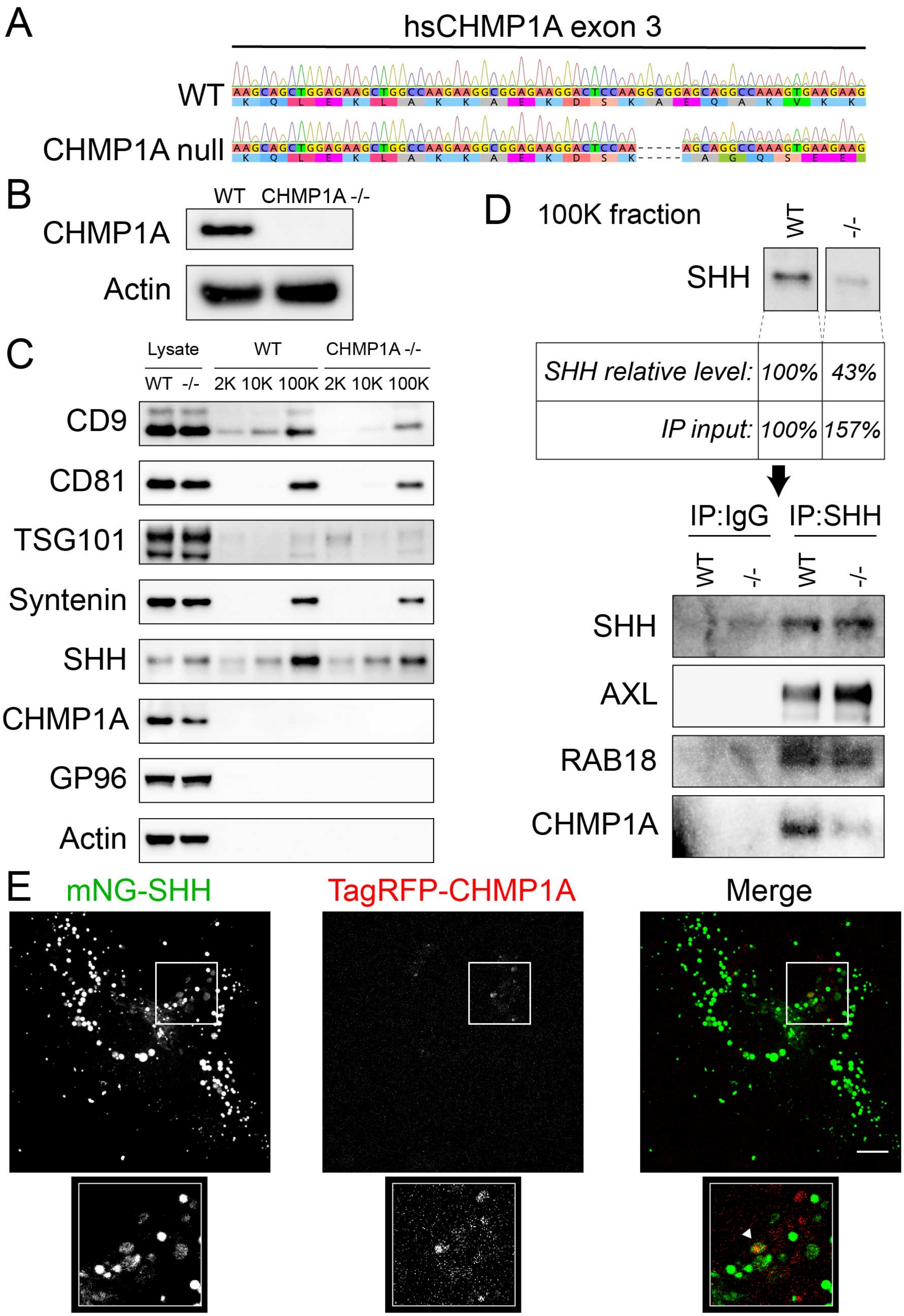

A
hsCHMP1A exon 3
WT
CHMP1A null
B
WT
CHMP1A -/-
CHMP1A
Actin
C
Lysate
WT
CHMP1A -/-
WT -/- 2K 10K 100K 2K 10K 100K
CD9
CD81
TSG101
Syntenin
SHH
CHMP1A
GP96
Actin
D
100K fraction
WT
-/-
SHH
SHH relative level: 100% 43%
IP input: 100% 157%
IP:IgG
IP:SHH
WT -/- WT -/-
SHH
AXL
RAB18
CHMP1A
E
mNG-SHH
TagRFP-CHMP1A
Merge

Figure S10. CHMP1A is associated with ART-EV complexes and partially co-distributes with intracellular SHH, Related to Figures 6 and 7

(A) *CHMP1A* null SVG-A cell line Sanger sequencing shows a 5 bp frameshift deletion in exon 3. This mutation creates a stop codon after exon 3. (B) No CHMP1A protein is detected by immunoblot in *CHMP1A* null SVG-A cells. (C) 100K EV fraction purified from pooled SVG-A cells transfected with *CHMP1A* null gRNA and Cas9. As we found in a stable monoclonal CHMP1A null SGV-A line, secretion of exosomal markers and SHH is decreased. (D) SHH-positive vesicles from the 100K pellet of WT and *CHMP1A* null SVGA cells were immunoisolated (right panel) using different input amounts (left panel), so that the SHH levels in the WT and KO input fractions are equivalent. WB analysis of the bound material shows CHMP1A in SHH-EVs from WT but not KO cells, while RAB18 and AXL are present in both fractions. (E) SVG-A cells were transfected with TagRFP-CHMP1A and mNG-SHH expressing plasmids and 48 h later, 15 z stacks of 0.5 µm were acquired every 5 seconds on a spinning disk confocal microscope using 488 and 561 nm lasers lines and associated filter sets. Snapshots show a z projection for each channel and the color-coded merge (upper panels). Cropped images (lower panels) show a single z plane, and the arrowhead points at colocalizing CHMP1A and SHH signals. Scale bar is 10 µm. See full video in **Movie S1**.
